# Progression of radial tears in L5-S1 intervertebral disc depends on location and type of movements: An in-silico study

**DOI:** 10.1371/journal.pone.0352680

**Published:** 2026-06-30

**Authors:** Vinyas, Subraya Krishna Bhat, Hiroshi Yamada, Raviraja Adhikari, Shyamasunder Bhat N

**Affiliations:** 1 Manipal Institute of Technology, Manipal Academy of Higher Education, Manipal, Karnataka, India; 2 Department of Biological Functions Engineering, Kyushu Institute of Technology, Kitakyushu, Japan; 3 Department of Mechanical Engineering, Manipal University Jaipur, Jaipur, Rajasthan, India; 4 Department of Orthopaedics, Kasturba Medical College, Manipal, Manipal Academy of Higher Education, Manipal, Udupi, Karnataka, India; Politecnico di Torino, ITALY

## Abstract

Low back pain is a global health challenge, with disc degeneration and annular tears contributing significantly to its onset and persistence. This numerical study evaluates how degeneration and the location of radial tears jointly influence the mechanical behavior of the L5–S1 spinal unit during flexion, extension, and lateral bending. A subject‑specific finite element (FE) model employing an anisotropic hyperelastic formulation was used to simulate healthy, mild, and moderate degeneration, along with radial tears spanning 75% of the annular width at four clinically relevant locations. Quantitative stress analysis revealed pronounced movement–tear location specificity, with posterior tears exhibiting approximately 4-6x higher boundary stresses during flexion, whereas anterior tears demonstrated 1.5-2x higher stresses during extension relative to other tear locations. Posterolateral (PL) tears exhibited their highest stresses during lateral bending, with PL1 (posterior‑shifted PL tear) showing 2-3x higher values and PL2 (more lateral PL tear) reaching 3-5x higher stresses compared to sagittal motions. Degeneration shifted the dominant stress contribution in PL2 from contralateral to ipsilateral lateral bending at the tear site, concurrently amplifying stress magnitudes in flexion and extension without altering the motion-dependent vulnerability patterns associated with each tear location. These findings offer biomechanical insights into how tear location and degeneration affect tear progression and may help guide clinicians in tailoring movement‑specific conservative or interventional treatment strategies.

## Introduction

Low back pain is a health problem of global relevance [1,2]. It is usually progressive in nature, which can disable the affected patients from performing their daily activities. Various factors, such as ageing, intense physical work, and poor posture, are responsible for this problem [3]. Degeneration of the disc due to ageing makes it brittle eventually causing it to tear [4]. This effect is reported to be the highest in the L5-S1 region [5]. The location and the wedge-shaped structural form of the disc in this spinal unit impose significantly high stresses when compared to the other regions of the spine [6]. This naturally puts the disc at a higher risk of tearing. Furthermore, mechanical overloading can enhance the chances of tear occurrence [7].

Internal disc disruptions or annular tears are often found to be the source of low back pain [8,9]. The recurrence of low back pain resulting from annular tears can possibly be reduced through early diagnosis and appropriate intervention [9]. Although annular tears have significant clinical relevance, computational investigations into their effects on spinal biomechanics remain limited.

**Fig 1:**
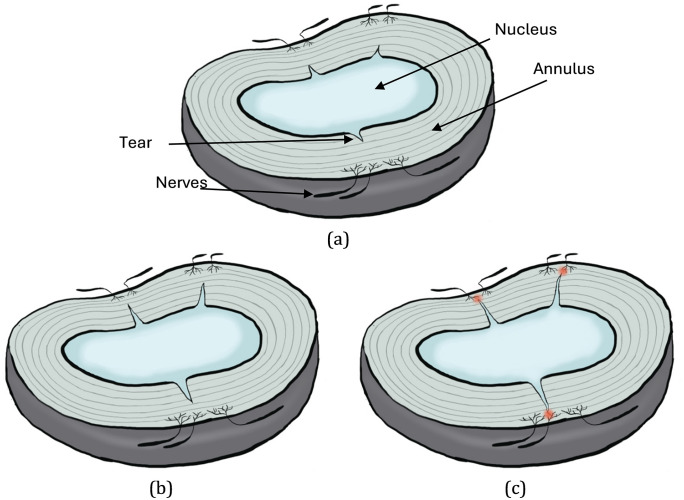
Annular radial tears, (a) initiation, (b) progression, (c) leading nuclear material to the innervations causing discogenic pain [11].

Radial tears are the second stage of internal disc disruptions, which form due to the coagulation of circumferential tears [10]. The progression of these tears is illustrated in Fig 1. They can direct the nucleus material to reach the highly innervated outer third of the annulus [12]. Also, in severe cases, the tears may surpass the annulus, causing conditions like herniation and sciatica. Gaining insight into the biomechanical changes caused by these tears can support the development of conservative treatments or other effective strategies to prevent further deterioration.

In-silico studies can provide information about the stress state of the IVD at any specific location of interest, which is useful for understanding the biomechanical response in different loading scenarios. This phenomenal advantage makes the approach popular for studying the spine and its associated ailments [13–15]. Many attempts have been made in the past to evaluate the effects of degeneration of the disc [13], but their focus was on the region above L5 vertebra, and generic models were used [13,16]. These models span from simplified representations to anatomically detailed constructs incorporating various tissue material properties and loading conditions. A recent review [16] provides a comprehensive summary of these advances, highlighting trends in modelling techniques and their clinical relevance.

Recent FE studies have modelled multiple lumbar levels and degenerative changes, particularly in the context of fusion instrumentation and adjacent‑segment mechanics at L5–S1 and across L1–S1 (e.g., comparative transforaminal lumbar interbody fusion cage sizing/placement at L5–S1 affecting ROM and endplate stress [17]; and whole‑lumbar dynamic analyses of degeneration effects and load re‑distribution from nucleus to annulus [18]). Similarly, multi‑level FE studies have examined topping‑off and fusion strategies with quantitative changes in ROM and disc/facet stresses, underscoring system‑level consequences of surgical decisions [19]. What remains underexplored, and is the central contribution of our study, is the explicit simulation of *radial annular tears* at clinically observed L5–S1 annulus locations, studied *together* with progressive annular material degeneration in a subject‑specific model, and quantified by stress at the *tear boundary* under physiologic motions.

While disc degeneration has been widely studied, recent computational investigations have focused on simulating progressive degeneration and its mechanical consequences. For instance, a 3D FE model of the L4-L5 segment was developed to simulate degenerative changes in the disc under mechanical stresses and range of motion [20]. Their findings suggested that avoiding certain movements and postures could help alleviate discogenic pain in patients suffering from disc degeneration. In another study [21], the dynamic characteristics of the L4-L5 segment with disc degeneration were evaluated. However, these studies have largely treated disc degeneration in isolation without explicitly modelling internal radial tears as a distinct pathological entity. The study by Little et al. [22] is particularly relevant to the present work, as it explored the effects of nucleus degeneration and radial tears at the L4-L5 level. Nonetheless, it excluded the vertebral bodies and degenerative changes in the annulus, which are critical aspects influencing disc mechanics. The current study builds on these foundations by being the first to simulate radial tear progression alongside annular material degeneration within the L5-S1 segment under physiologically relevant loading. In contrast to prior studies, our work interrogates how tear *location* interacts with *movement type* (flexion, extension, lateral bending) and *degeneration level* to amplify stresses in the tear boundary revealing motion and location‑dependent stress patterns.

The current study includes radial tears as they are commonly found in the lower region of the spine [23,24]. These tears are commonly present in the posterior and posterolateral regions of the annulus [25]. The posterolateral tear is of particular significance as it is also a common location for the appearance of herniation, which is a consequence of radial tear growth [26]. An anterior radial tear is included, as radial tears are postulated to be a consequence of coagulation of circumferential tears and they are commonly observed in the anterior region [10,25]. Our study intends to evaluate the state of IVD due to the degeneration of the annulus and nucleus in the presence of a radial tear under sagittal and lateral movements.

A subject-specific 3D model is employed with the representation of the annulus by the Gasser-Ogden-Holzapfel (GOH) material model [11,14,27]. The L5-S1 level of the spine is chosen for the study, which has received less attention in the computational research domain despite its major clinical significance. Also, the effects of degeneration on the annulus and the nucleus are considered. To date, no finite element studies have examined the L5-S1 disc segment with simultaneous consideration of radial annular tears and progressive annular material degeneration. Moreover, most existing degeneration models neglect the structural behavior of annular material. This study addresses these gaps by incorporating annular degeneration using a structurally motivated Holzapfel-Gasser-Ogden (HGO) model, enabling more physiologically accurate representation of the annulus. The L5-S1 level, being the most mechanically loaded and clinically vulnerable disc, warrants this detailed investigation.

## Method

### FE modelling

**Fig 2:**
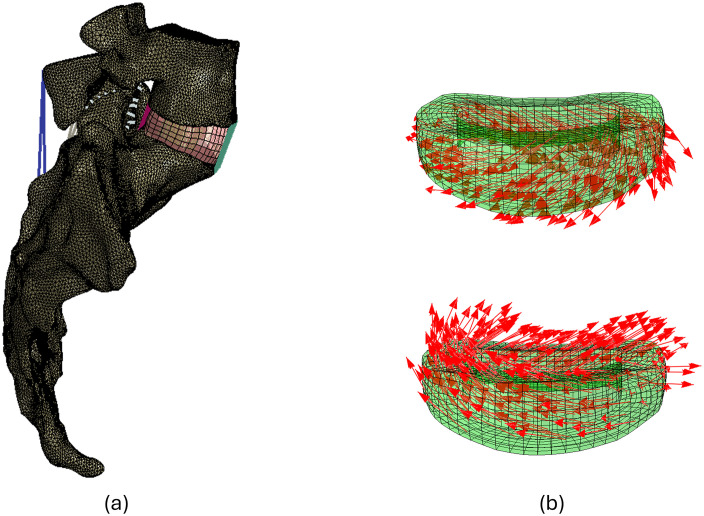
(a) FE model of L5-S1 spinal unit (b) Fiber orientation indicated by arrows [11].

The Institutional Ethics Committee (IEC) of Kasturba Medical College and Kasturba Hospital has granted institutional approval [IEC Project Number IEC:931/2018]. This study employed a previously validated finite element (FE) model of an intact L5-S1 spinal segment, as shown in Fig 2a. The annulus model was modified to include tears. The vertebral bodies, endplates, and nucleus pulposus were constructed based on well-established modelling approaches [14]. As the study is primarily focused on the annulus, its structural characteristics are briefly revisited. Fig 2b illustrates the fiber orientation within the annulus matrix in the FE model, where arrows represent the fiber alignment at ± 30˚ relative to the horizontal plane.

The annulus fibrosus was meshed using 8-node hybrid brick elements (C3D8H) in ABAQUS CAE® (Dassault Systèmes), which ensured less sensitivity to the mesh size. 8 elements each along the axial and radial directions were adopted as a result of the mesh convergence test. This mesh configuration ensured computational efficiency and stress accuracy, as validated in previous studies [14].

**Fig 3:**
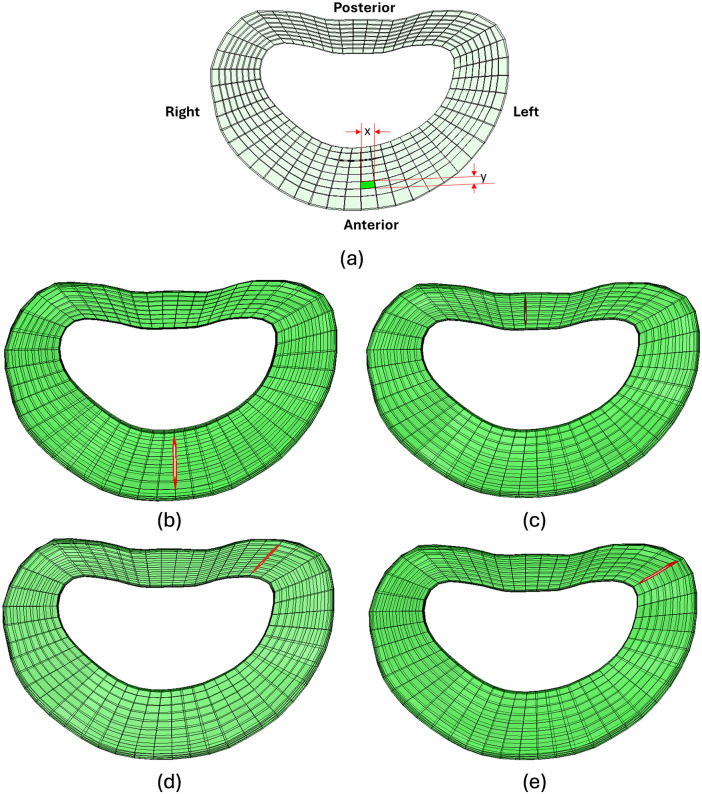
a) Scale bar of annulus top view with dimensions measuring x = 1.6 mm and y = 0.9 mm. Top view of the annulus with radial tear at the, b) anterior, c) posterior, d) left posterolateral type 1 (PL 1), e) left posterolateral type 2 (PL 2).

To simulate radial tears, elements within the annulus were duplicated, and their nodes were displaced using the morphing tools available in HyperMesh (HyperWorks, Inc.). This approach allowed for the introduction of tears while retaining the original shape and structural integrity of the annulus [11,28]. Fig 3a shows the annulus with a scale bar to assess the relative size of the tears. A total of four radial tears as shown in Fig 3b–3e were introduced: two located posterolaterally (PL1 and PL2), one anteriorly, and one posteriorly. Each tear extended through six of the eight radial elements, corresponding to approximately 75% of the annular thickness. Though not based on a specific clinical threshold, the length was intentionally chosen to reflect substantial structural disruption and to illustrate the biomechanical consequences of such tears.

### Material model and boundary conditions

A subject-specific finite element model of the L5–S1 spinal unit was used to investigate the biomechanical influence of radial tears and degeneration. The annulus fibrosus was modeled using the Gasser-Ogden-Holzapfel (GOH) hyperelastic formulation, which captures its anisotropic and nonlinear mechanical characteristics. This approach is supported by Gruber et al. [29], who demonstrated that the GOH model offers superior physiological fidelity and computational robustness when simulating annular tissue response to complex loading conditions. This continuum-based representation avoids the limitations of discrete fiber modelling and allows for a more physiologically realistic simulation of annular mechanics under load.

In modelling annular degeneration, we employed the systematic approach previously developed and validated by the authors in [11]. Experimental studies have shown that the annulus fibrosus stiffens substantially with degeneration, with increases of up to 4.7‑fold in Young’s modulus of the ground matrix reported for severely degenerated discs [3,30,31]. Because the annulus is a nonlinear, anisotropic material, adjusting only the Young’s modulus is insufficient to capture degeneration‑induced stiffening across the full strain range. Therefore, we analytically derived the initial tangent modulus (ITM) of the GOH model under equibiaxial stretch, allowing controlled modification of the small‑strain stiffness independently of other parameters. To represent the experimentally observed stiffening at larger strains, we adjusted the nonlinear parameter *k₂*, which strongly influences the curvature of the stress–strain response at higher deformation levels. In accordance with published experimental data and our earlier modelling work, mild and moderate degeneration were simulated by scaling the ITM and increasing *k₂* by 50% and 100%, respectively. This combined ITM–*k₂* adjustment yields a physiologically consistent representation of degeneration‑induced stiffening throughout the entire strain regime.

The material properties for other components of the spinal unit, including vertebral bones, nucleus pulposus, and ligaments, were adapted from established literature sources and are summarized in Table 1. The baseline properties of the healthy nucleus pulposus were taken from Guan et al. [29], who provide widely used elastic modulus and Poisson’s ratio values for intact disc models. Although this reference does not model degeneration directly, experimental studies suggest that the nucleus becomes mechanically similar to the annulus ground matrix during degeneration due to increased solid‑phase stiffness and reduced fluid content [3]. Consistent with our previous work [11], and to reflect this progressive stiffening, the Young’s modulus of the nucleus was increased proportionately to the changes applied to the annulus, resulting in values of 2 MPa (mild) and 3 MPa (moderate).

**Table 1:** Material properties and element types used for vertebra, nucleus, annulus and ligaments involved in the in-silico model.

Part name	Element type	Young’s Modulus (MPa)	Poisson’s ratio
Cortical [32]	C3D4	12000	0.3
Cancellous [31]	C3D4	200	0.3
Nucleus [11,33]	C3D8H	1 [Healthy]	0.499
		2 [Mild]	0.499
		3 [Moderate]	0.499
Annulus [11]		C_1_ = 0.4323 MPa, k_1_ = 2.1638 MPa, k_2_ = 200 [Healthy]	
		C_1_ = 0.75 MPa, k_1_ = 3.5 MPa, k_2_ = 300 [Mild]	
		C_1_ = 1 MPa, k_1_ = 5 MPa, k_2_ = 400 [Moderate]	
Ligaments [34]	3D Truss element	Hypoelastic constitutive law (Tension only), Incremental elastic modulus in MPa (Strain %)	
Anterior Longitudinal Ligament		7.8 (ε<12%), 20 (ε>12%)	
Posterior Longitudinal Ligament		10 (ε<10%), 20 (ε>10%)	
Ligamentum Flava		15 (ε<6%), 19.5 (ε>6%)	
Interspinous Ligament		10 (ε<15%), 11.6 (ε>15%)	
Supraspinous Ligament		8 (ε<20%), 15 (ε>20%)	
Capsular Ligament		7.5 (ε<25%), 30(ε>25%)	

Boundary conditions were designed to simulate four physiological movements: flexion, extension, left lateral bending, and right lateral bending. A bending moment of 4 Nm was applied to a central reference point on the superior endplate of the L5 vertebra in all simulations [35]. This reference point was kinematically coupled with the nodes on the upper surface of L5 to ensure uniform moment distribution. The sacrum (S1) was constrained by fixing its centroid, which was connected to all sacral nodes via multi-point constraints (MPCs), following the approach in previous studies [11,14]. This magnitude was selected based on our previously validated L5–S1 model [14], which demonstrated that the annulus fibrosus exhibits strongly non‑linear behaviour in the low‑moment region (up to ~4 Nm), with progressive fibre recruitment and rapid changes in stiffness. Because the objective of this study was to examine the mechanical response of radial tears and degeneration within this non‑linear regime, where tear opening, local shear concentration, and degeneration‑dependent stiffening are most pronounced, a moment load of 4Nm was used. Loading beyond 4 Nm shifts the annulus toward a more linear mechanical response, reducing sensitivity to these localised effects. Hence, 4 Nm provides a more appropriate loading condition for evaluating the comparative mechanical behaviour of tears and degenerative states.

### Shear‑stress based Tresca evaluation criterion

The maximum shear stress failure criteria (Tresca criterion) was used in its evaluation as shearing has a significant contribution to causing damage [14,36,37]. The Tresca maximum shear stress criterion was adopted primarily because shear stress is known to be predominant between the lamellae of the annulus fibrosus and plays a key role in delamination and fiber-matrix separation, particularly around regions of internal tearing [38]. Additionally, Tresca stress effectively captures the localized shearing at the surface of the tear, making it well-suited for assessing annular damage from a structural standpoint.

Although Tresca stress provides a useful measure of localized shear amplification, particularly near tear tips, the ultimate shear strength of annulus fibrosus tissue is not well characterized in the literature, and available failure data do not distinguish between healthy, mildly degenerated, and moderately degenerated states. Published annulus fibrosus failure metrics are typically obtained under uniaxial tension along the fiber direction, which differs substantially from the multiaxial shear‑dominated state modelled here. For these reasons, the present study interprets Tresca stress comparatively rather than in absolute relation to tissue failure thresholds.

## Results

**Fig 4:**
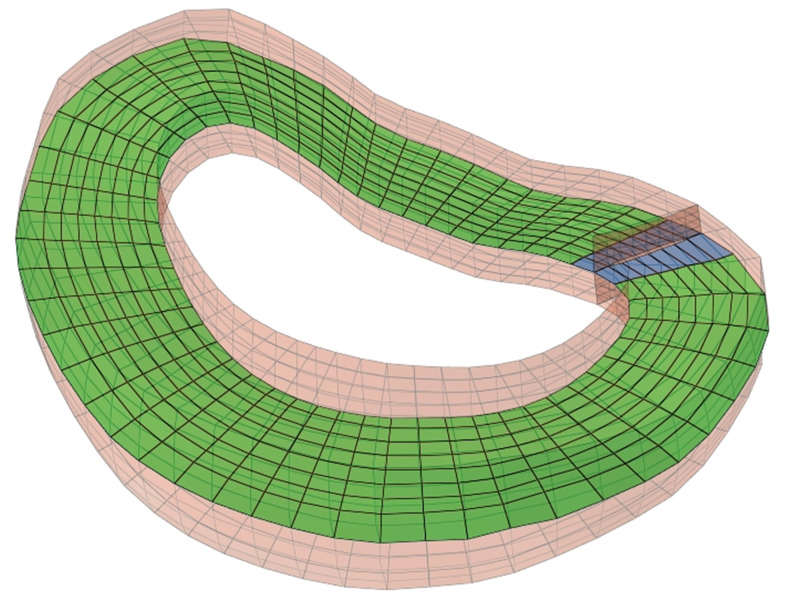
The mid-plane of the annulus represented with opacity.

The information on the location of stress concentration may provide biomechanical insight into regions of increased structural vulnerability within the intervertebral disc, which can complement clinical understanding of discogenic pain, without implying a direct or sole prediction of pain based on mechanical measures alone [39]. The mid-plane of the annulus represented as opaque in Fig 4, was used to avoid numerical errors resulting from the sharp difference in the stiffness at the interface of the vertebra and the disc [40,41].

### Effect of location of tears and degeneration during flexion

**Fig 5:**
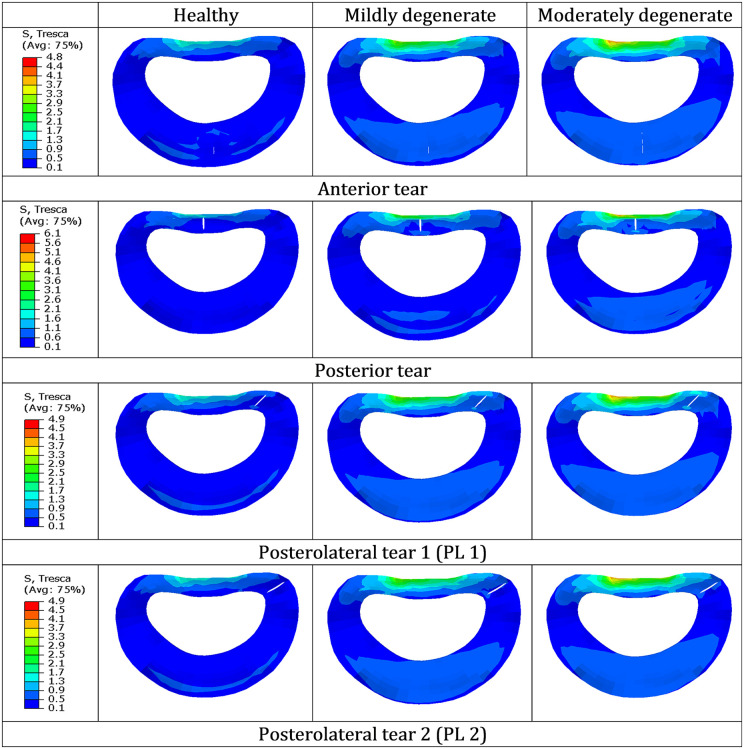
Mid-plane Tresca shear stresses in healthy and degenerate cases of annulus with different radial tears for flexion.

**Fig 6:**
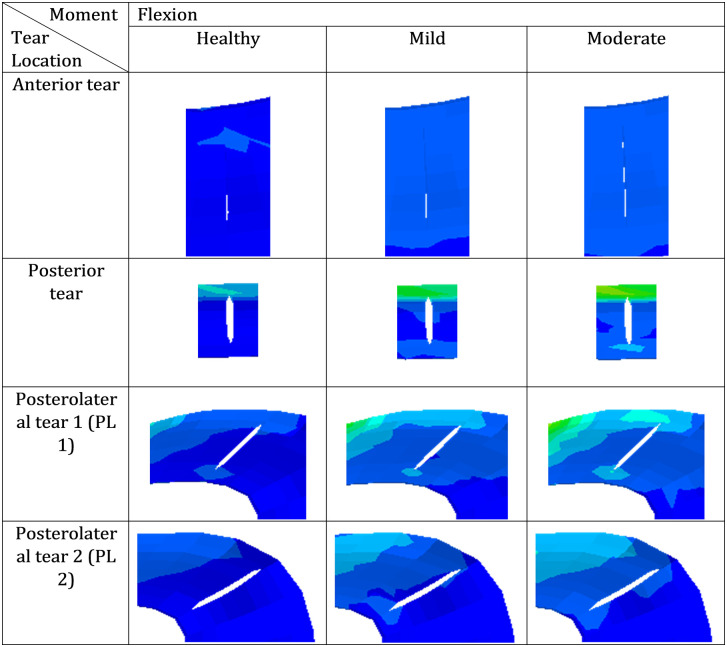
Magnified view of tears shown in Fig 5.

**Fig 7:**
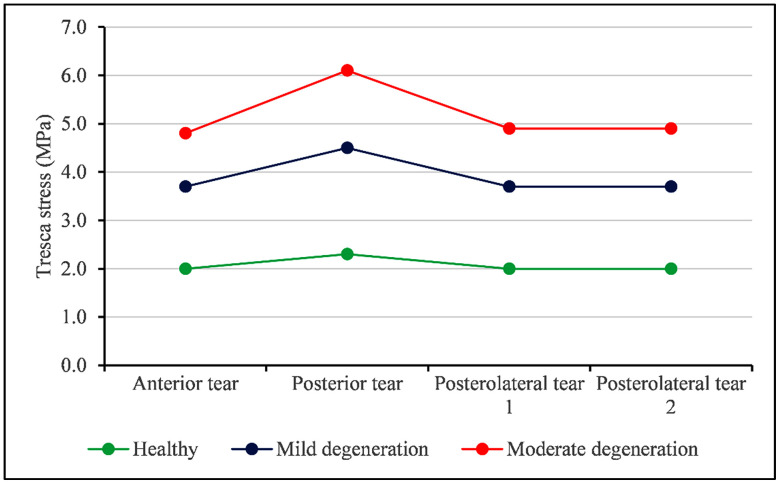
Graphical representation of maximum mid-plane Tresca stress for the conditions addressed in flexion.

According to the stress contour in Fig 5, among the four cases of tears considered for this study, the anterior tear produced minimal deviation from the healthy‑annulus stress pattern, indicating that its influence is small relative to the other tear locations. The posterolateral tears caused some stress relaxation near the site of the tear. The direct posterior tear in case 2 was found to be critical during flexion presenting higher stress concentration in the posterior region for all cases of degeneration compared to other locations. The close-up view of the tears in Fig 6, shows that except for the anterior tear, the rest of the tears in the posterior and posterolateral tended to open. Fig 7, shows the variation of maximum Tresca stress with degeneration for the different tears.

The maximum mid-plane Tresca stress increased with degeneration and was highest for the posterior tear, rising from approximately 2.3 MPa in the healthy model to 4.5 MPa in the mildly degenerated model and reaching a peak of 6.1 MPa in the moderately degenerated model. The anterior, PL1, and PL2 tears produced comparable mid-plane stress values of approximately 2 MPa, 3.7 MPa, and 4.9 MPa for the healthy, mildly degenerated, and moderately degenerated states, respectively. These values were consistently lower than those observed at the posterior tear, and this difference became more pronounced with increasing degeneration.

### Effect of location of tears and degeneration during extension

**Fig 8:**
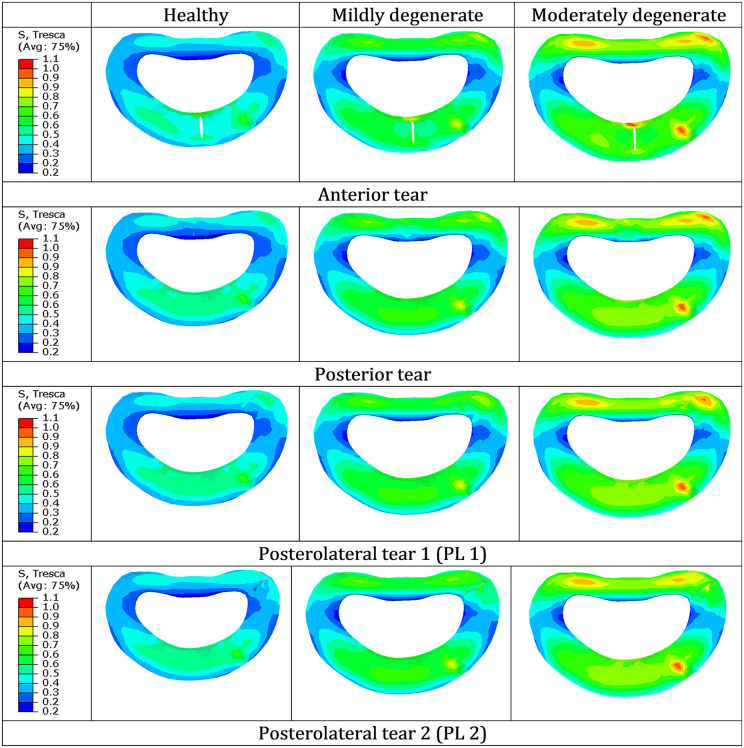
Mid-plane Tresca shear stresses in healthy and degenerate cases of annulus with different radial tears for extension.

**Fig 9:**
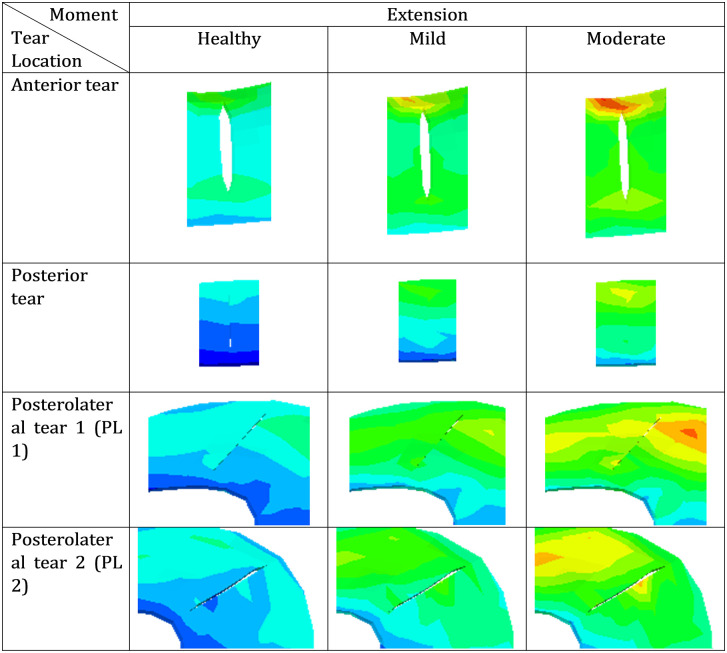
Magnified view of tears shown in Fig 8.

**Fig 10:**
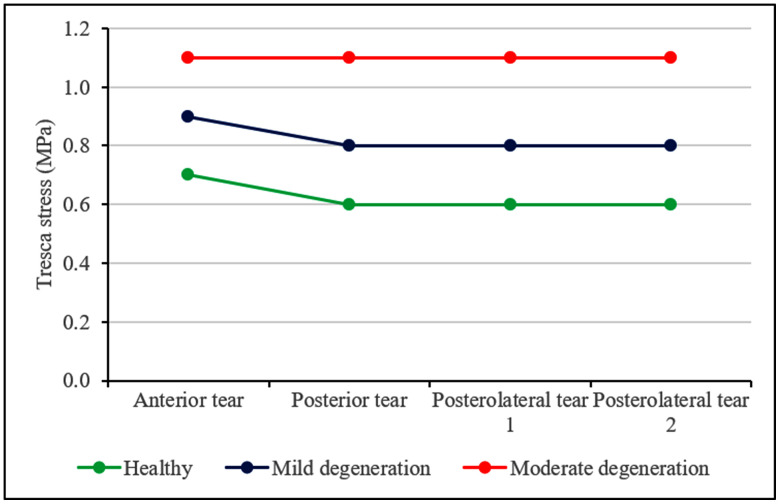
Graphical representation of maximum mid-plane Tresca stress for the conditions addressed in extension.

The effect of location of the tear was opposite to that for flexion. The radial tear in the anterior location seemed to be perturbing compared to other locations. Localized stress concentration and opening of the tear were visible in the case of anterior tear for all levels of degeneration as seen in both Figs 8 and 9. Independent of the location of tears, a rise in stress was observed in the posterior region (refer to Fig 8) of the annulus with an increase in the level of degeneration. The posterior and posterolateral tears tended to close without any significant stress variations for healthy annulus, whereas, in the case of degeneration, the stress concentration zone appeared to overlap with the location of the tear. Fig 10, shows the variation of maximum Tresca stress in the mid-plane with degeneration for the different tears. Under extension loading, the mid-plane stress in the presence of an anterior tear was marginally elevated compared to the remaining tear locations. Specifically, values of 0.7 MPa and 0.9 MPa were observed in the healthy and mildly degenerated states, respectively, while the posterior and posterolateral tears uniformly recorded 0.6 MPa and 0.8 MPa under the corresponding conditions. In the moderately degenerated state, the peak mid-plane stress value was 1.1 MPa for all tear locations, indicating that the effect of tear position on mid-plane stress becomes negligible at advanced degeneration levels.

The anterolateral stress peak appears as a localized anomaly associated with geometric variations at the endplate–annulus interface which may be further amplified by the innate wedge-shaped geometry of the intervertebral disc. In addition, asymmetric loading characterized by anterior tensile and posterior compressive stresses contributes to this effect, which remains distinct from tear-induced stress amplification.

### Effect of location of tears and degeneration during left lateral bending

**Fig 11:**
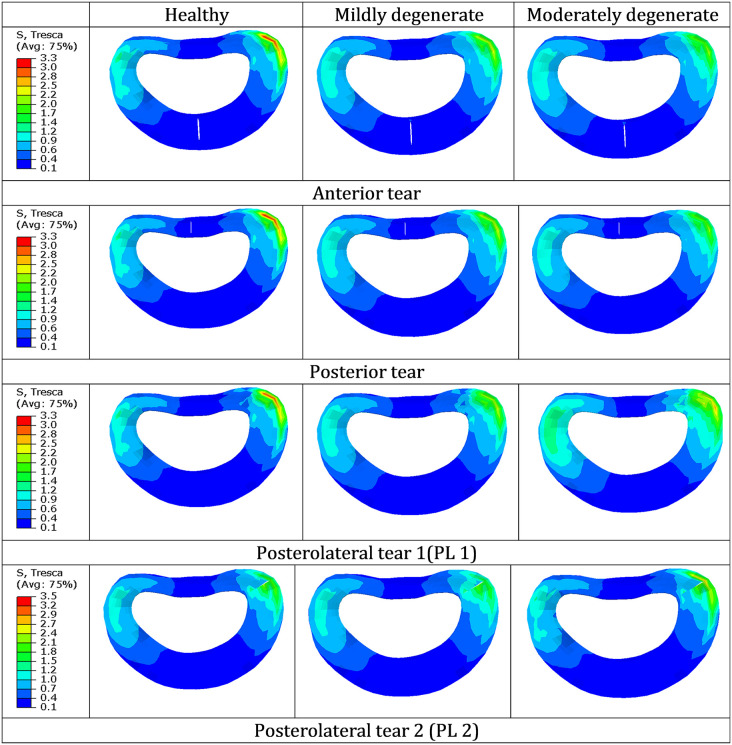
Mid-plane Tresca shear stresses in healthy and degenerate cases of annulus with different radial tears for left lateral bending.

**Fig 12:**
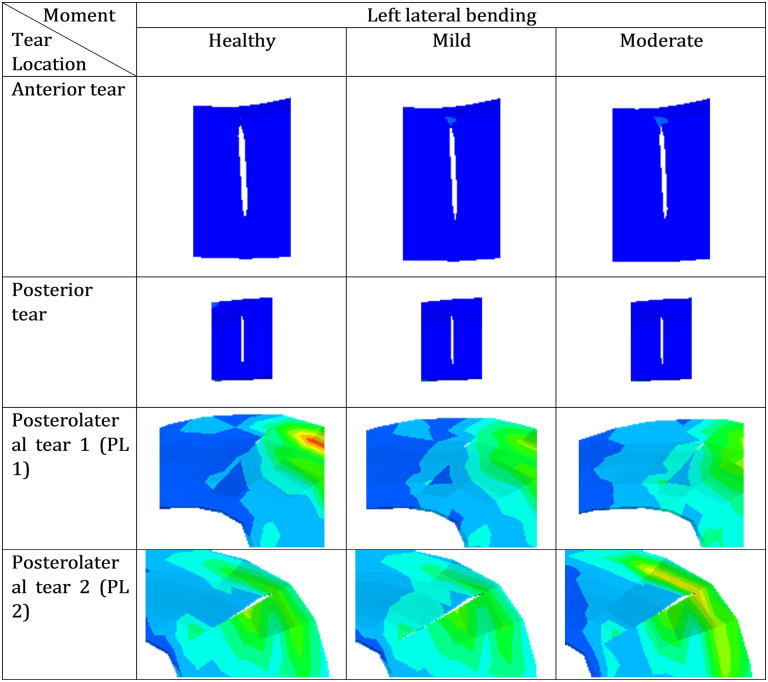
Magnified view of tears shown in Fig 11.

The introduction of a tear altered the characteristic stress distribution observed in a healthy annulus. The anterior tear and posterior tear did not produce significant local changes in the stress during the lateral bending moment (refer to Fig 11). The posterolateral tears tended to close with high stress concentrated towards the posterolateral side of the annulus. The stress concentration affected PL2 to a greater extent than PL1 as observed in Fig 12. It indicates that the left lateral bending can cause worsening of the posterolateral tears, which are aligned more laterally compared to the posterolateral tears, which are aligned towards the posterior.

**Fig 13:**
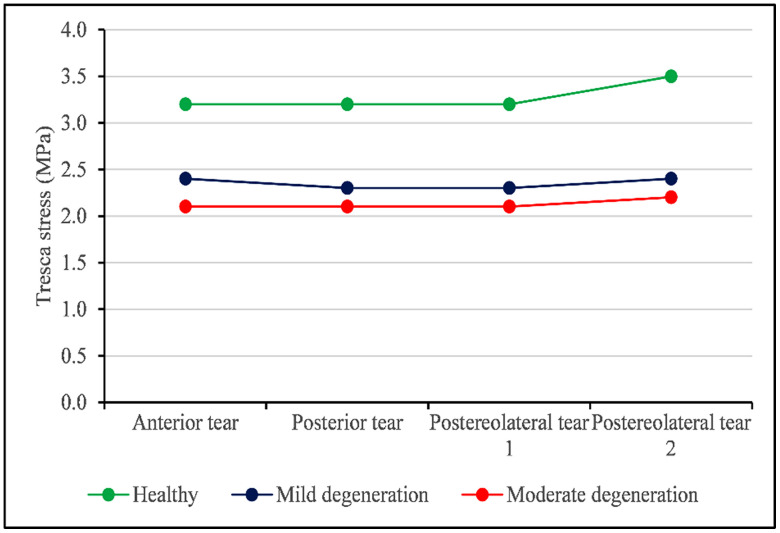
Graphical representation of maximum mid-plane Tresca stress for the conditions addressed in left lateral bending.

Fig 13, shows the variation of maximum Tresca stress with degeneration for the different tears. Unlike flexion and extension, the maximum mid-plane Tresca stress decreased with degeneration. This reduction is attributed to the restriction of segmental motion caused by degeneration-induced annular stiffening, which limits the range of movement and consequently reduces the stress experienced at the mid-plane. The highest mid-plane stress was observed in the healthy state, where the anterior, posterior, and PL1 tears showed comparable values of approximately 3.2 MPa, while PL2 exhibited a marginally greater stress of 3.5 MPa. With increasing degeneration, the mid-plane stress decreased considerably to approximately 2.4 MPa and 2.1 MPa under mild and moderate degeneration, respectively, across all tear locations.

### Effect of location of tears and degeneration during right lateral bending

**Fig 14:**
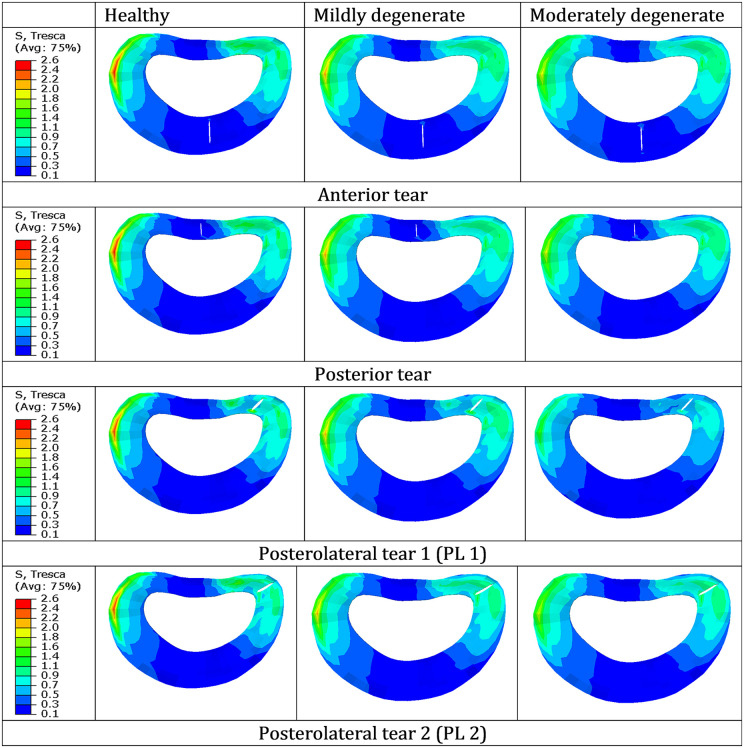
Mid-plane Tresca shear stresses in healthy and degenerate cases of annulus with different radial tears for right lateral bending.

**Fig 15:**
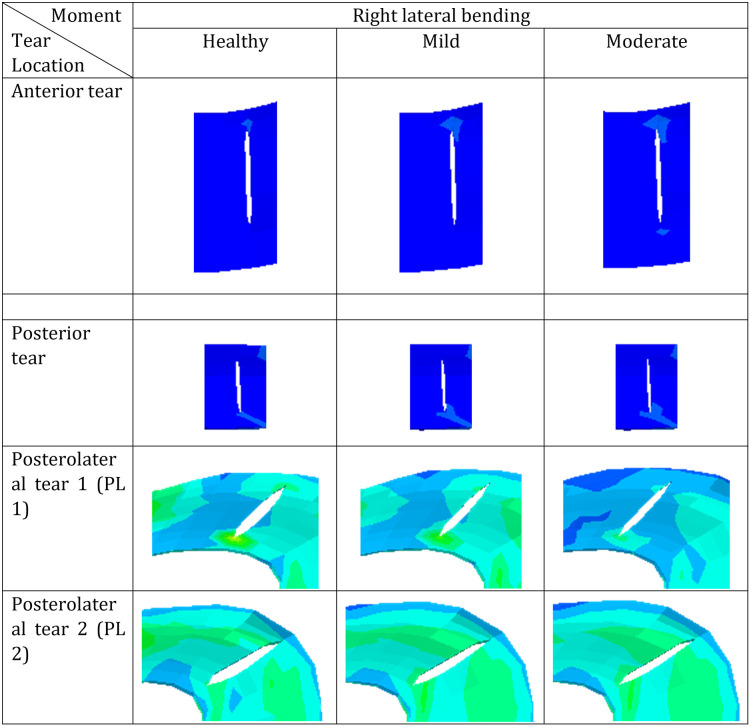
Magnified view of tears shown in Fig 14.

**Fig 16:**
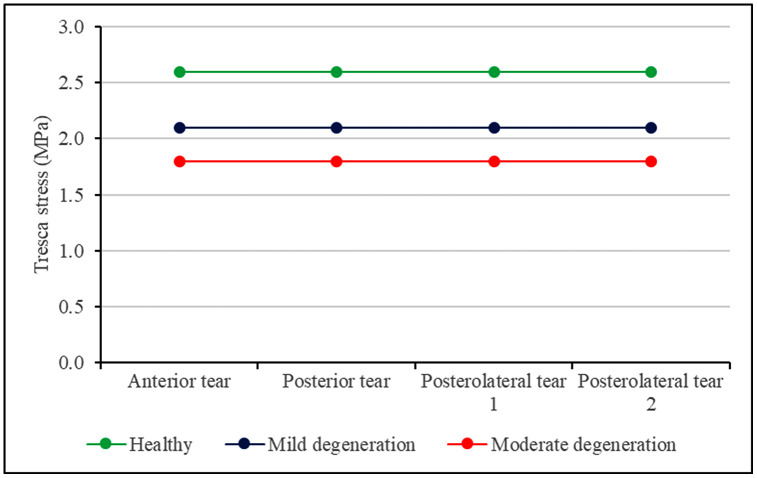
Graphical representation of maximum mid-plane Tresca stress for the conditions addressed in right lateral bending.

The stress pattern compared to the intact model followed a similar trend to the healthy‑annulus configuration described for left lateral bending. The stress patterns for right lateral bending (refer to Fig 14) appeared to be mirror images of the stress patterns obtained for left lateral bending in the case of annulus with radial tears. Fig 15 shows the nature of the deformation of the tear in the case of posterolateral tears was opposite to that of left lateral bending. The tears tended to open, complemented by a high-stress region around the tear and at the tip of the tear. The extent of deformation of the tear in a healthy state was evidently high compared to tears in a degenerated annulus. Fig 16, shows the variation of maximum mid-plane Tresca stress with degeneration for the different tears. The trend of stresses for the different positions of tears was similar across both lateral bending movements, with peak mid-plane Tresca stress values of approximately 2.6 MPa (healthy), 2.1 MPa (mild), and 1.8 MPa (moderate degeneration). The midplane stress values were independent of the tear positions.

### Polar plots to present variation of stress at the tear boundary

**Fig 17:**
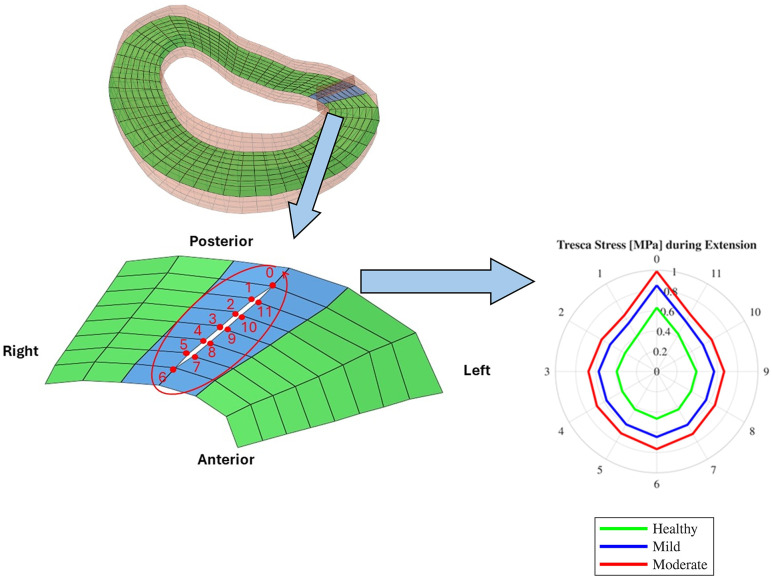
Sample figure showing relation between the nodes and the points on the polar plot.

The effect of (a) level of degeneration, (b) location, and (c) movement on the stress state at the tear boundary is assessed using polar plots. Fig 17 illustrates the correspondence of nodes along the tear boundary to the plotted points. This method was used in all the other cases as well.

### Effects of degeneration from polar plots

**Fig 18:**
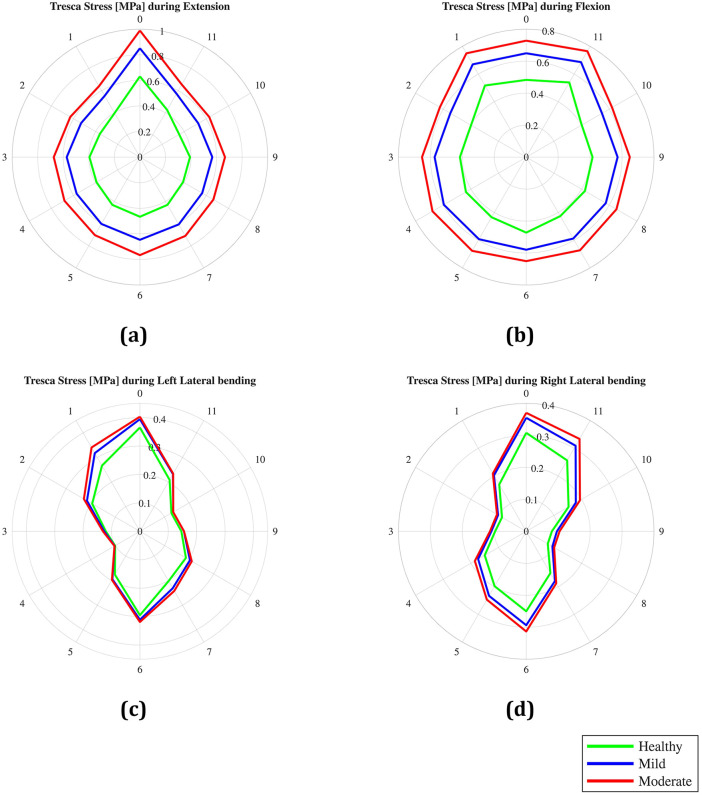
Polar plot of Tresca stress [MPa] for various degeneration levels in (a) Extension, (b) Flexion, (c) Left lateral bending and (d) Right lateral bending at the boundary of the anterior radial tear.

**Fig 19:**
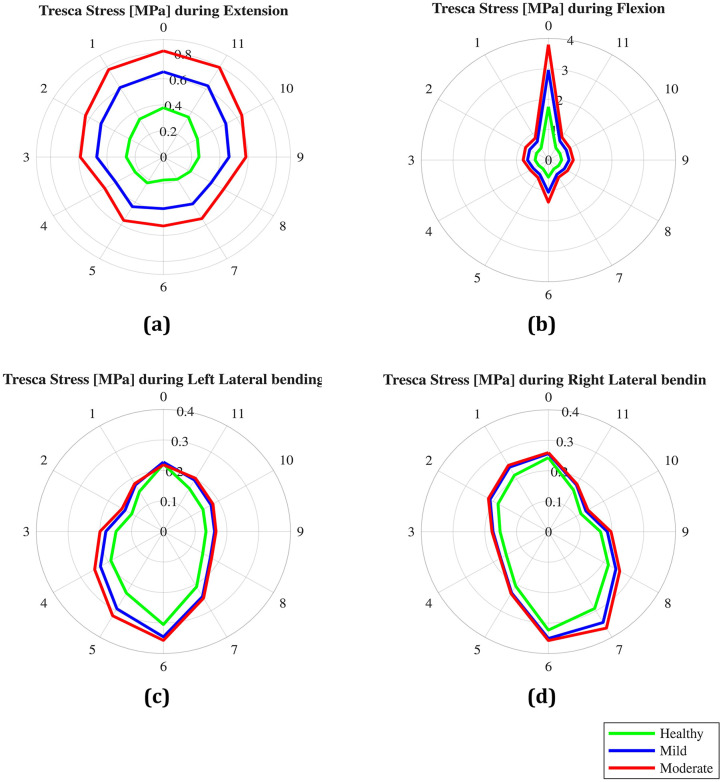
Polar plot of Tresca stress [MPa] for various degeneration levels in (a) Extension, (b) Flexion, (c) Left lateral bending and (d) Right lateral bending at the boundary of the posterior radial tear.

**Fig 20:**
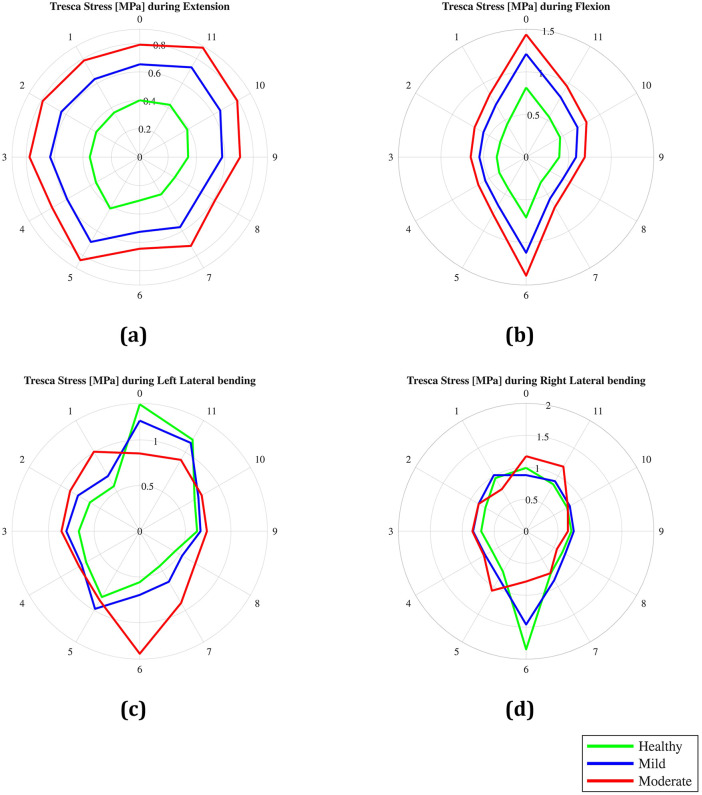
Polar plot of Tresca stress [MPa] for various degeneration levels in (a) Extension, (b) Flexion, (c) Left lateral bending and (d) Right lateral bending at the boundary of the Posterolateral radial tear (PL1).

**Fig 21:**
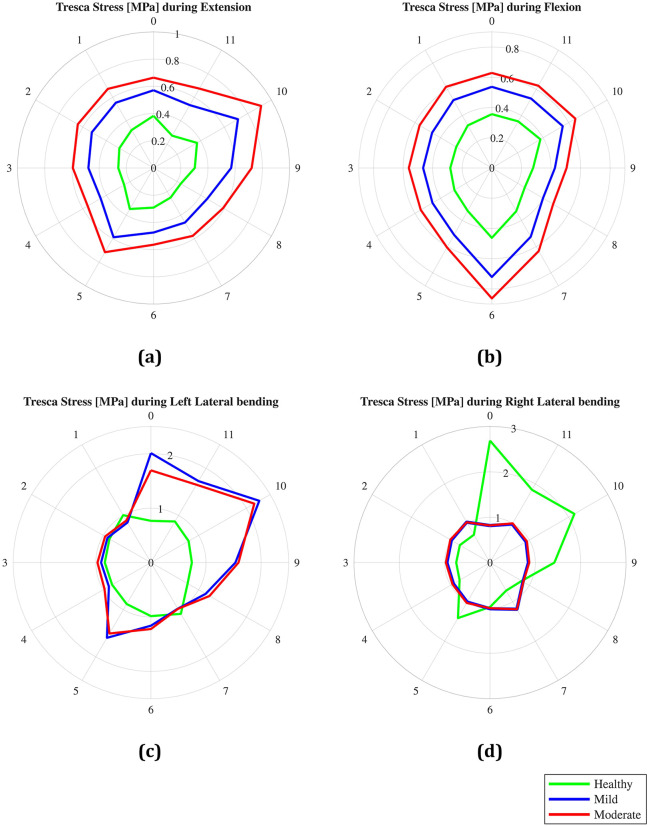
Polar plot of Tresca stress [MPa] for various degeneration levels in (a) Extension, (b) Flexion, (c) Left lateral bending and (d) Right lateral bending at the boundary of the Posterolateral radial tear (PL2).

Figs 18–21 show the effect of degeneration on Tresca stress variations in the tear boundary for anterior, posterior, PL1 and PL2, respectively, during four physiological movements. For the posterior tear under flexion (Fig 19b), the peak boundary Tresca stress reached approximately 2.0–4.0 MPa across all degeneration states, reflecting consistently high susceptibility at this location regardless of tissue quality. For the anterior tear under extension (Fig 18a), the peak boundary stress was approximately 1.0 MPa in the denerated model, reducing slightly to ~0.8 MPa in the healthy models. For PL2 under left lateral bending (Fig 21c), an exception was observed: the peak boundary stress was approximately 2.0 MPa across mild and moderate degeneration, compared to near-zero values in the healthy state, indicating that degeneration amplifies the vulnerability of this tear specifically under left lateral bending

This effect was negligible on the boundaries of anterior and posterior tears for lateral movements. An exception was observed for PL2, where left lateral bending produced a marked increase in boundary stresses under mild and moderate degeneration compared with the healthy annulus. Notably, with progressive degeneration, stress magnitudes at the same tear boundary decreased under right lateral bending.

### Effects of location of tears from polar plot

**Fig 22:**
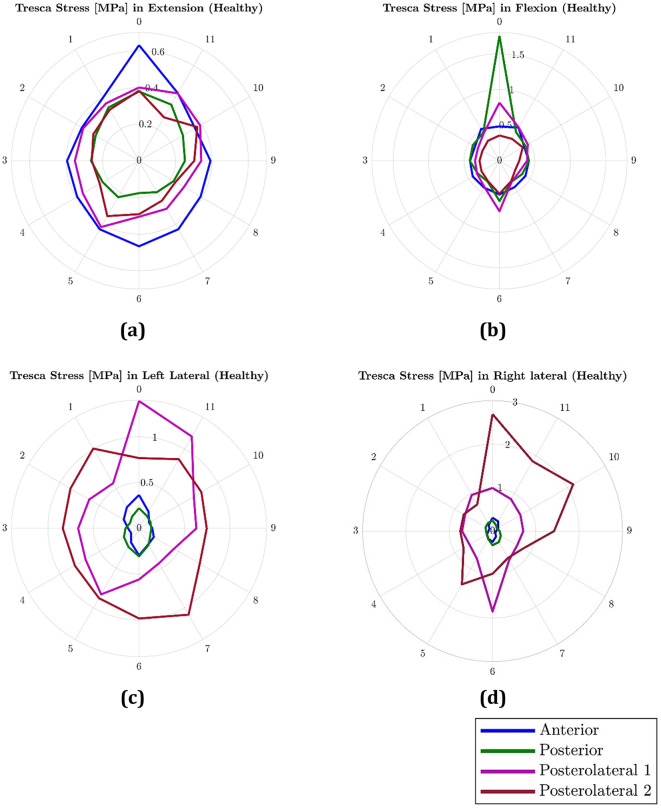
Tresca stress polar plots at the tear boundary of the healthy annulus with varying location of tears for different movements: (a) Extension, (b) Flexion, (c) Left lateral bending and (d) Right lateral bending.

**Fig 23:**
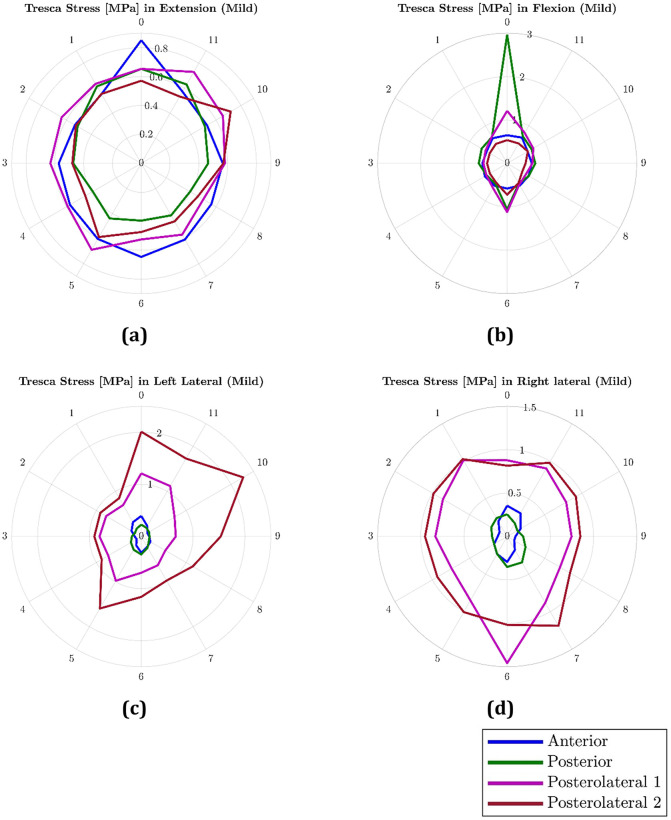
Tresca stress polar plots at the tear boundary of the mildly degenerated annulus with varying location of tears for different movements: (a) Extension, (b) Flexion, (c) Left lateral bending and (d) Right lateral bending.

**Fig 24:**
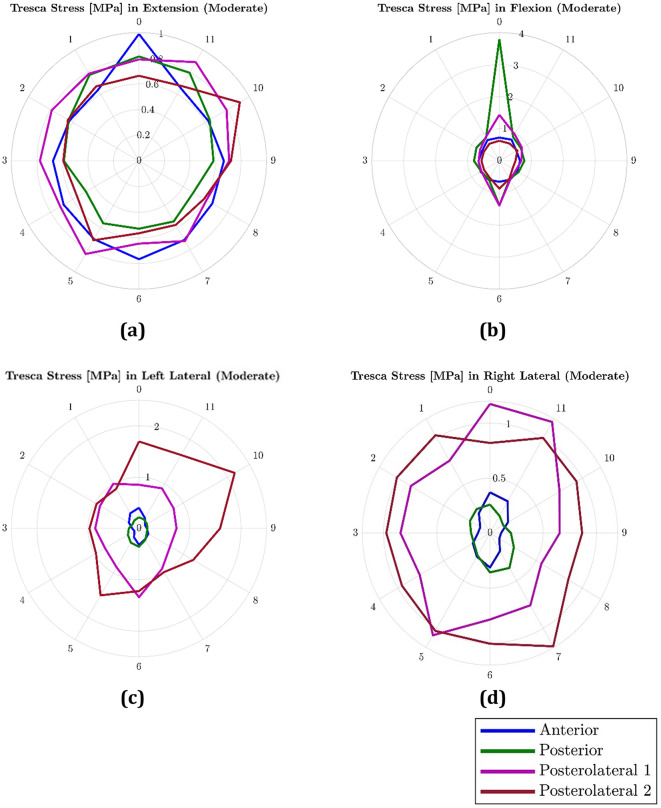
Tresca stress polar plots at the tear boundary of the moderately degenerated annulus with varying location of tears for different movements: (a) Extension, (b) Flexion, (c) Left lateral bending and (d) Right lateral bending.

Figs 22–24 show the effect of location of tears on Tresca stress variations in the tear boundary for healthy, mild and moderate levels of material degeneration levels, respectively, during four physiological movements. Across all degeneration states, posterior tears are most vulnerable during flexion, with stress magnitudes 4-6x higher than any other tear location. In contrast, anterior tears dominate during extension, reaching values around 1.5-2x those of the posterior and posterolateral tears under degeneration. For lateral bending, PL2 repeatedly shows the highest susceptibility, with stress magnitudes on the order of 4-8x greater than anterior/posterior tears and 2-3x greater than PL1, consistently across healthy, mild, and moderate degeneration. Degeneration amplifies stress in flexion and extension but does not change the fundamental ranking of vulnerability dictated by tear location and motion type.

### Effects of movements on Tresca stress from polar plots

**Fig 25:**
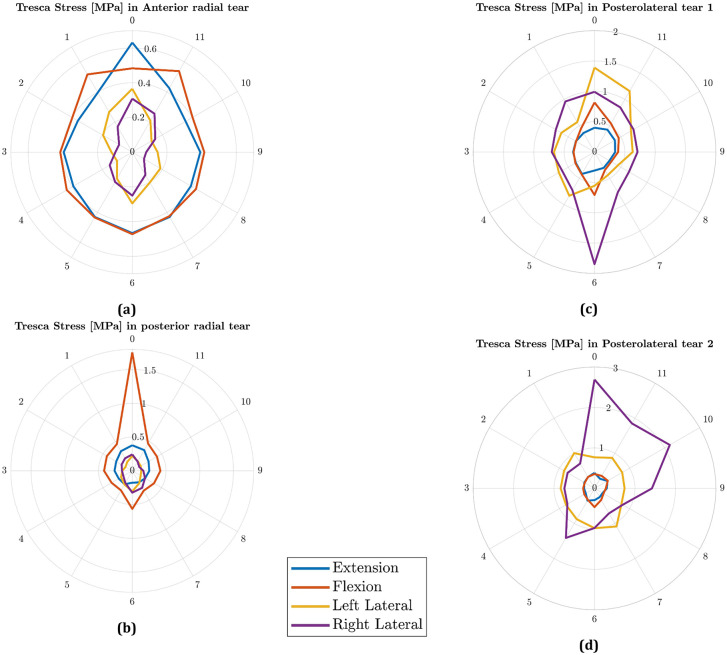
Polar plot for effects of movements on the boundary of (a) Anterior radial tear, (b) Posterior radial tear, (c) Posterolateral tear 1, (d) Posterolateral tear 2, in healthy annulus.

**Fig 26:**
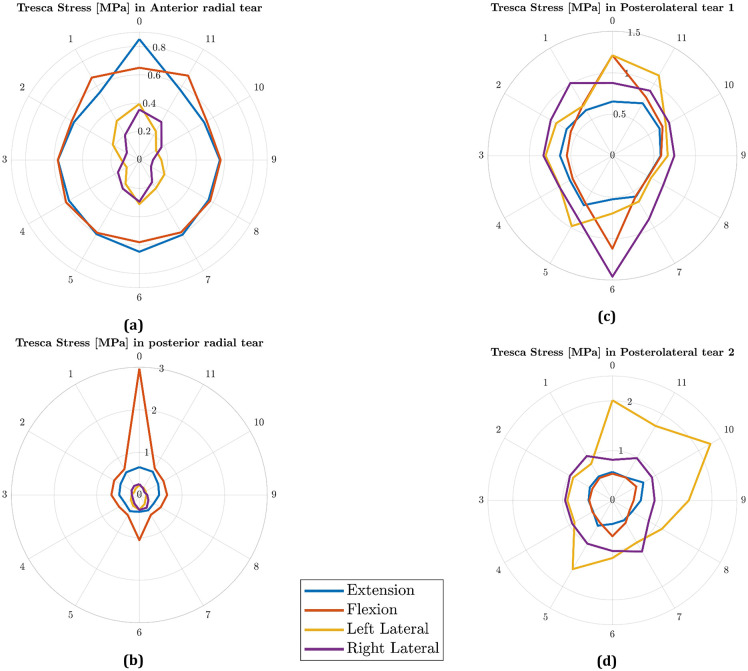
Polar plot for effects of movements on the boundary of (a) Anterior radial tear, (b) Posterior radial tear, (c) Posterolateral tear 1, (d) Posterolateral tear 2, in mildly degenerated annulus.

**Fig 27:**
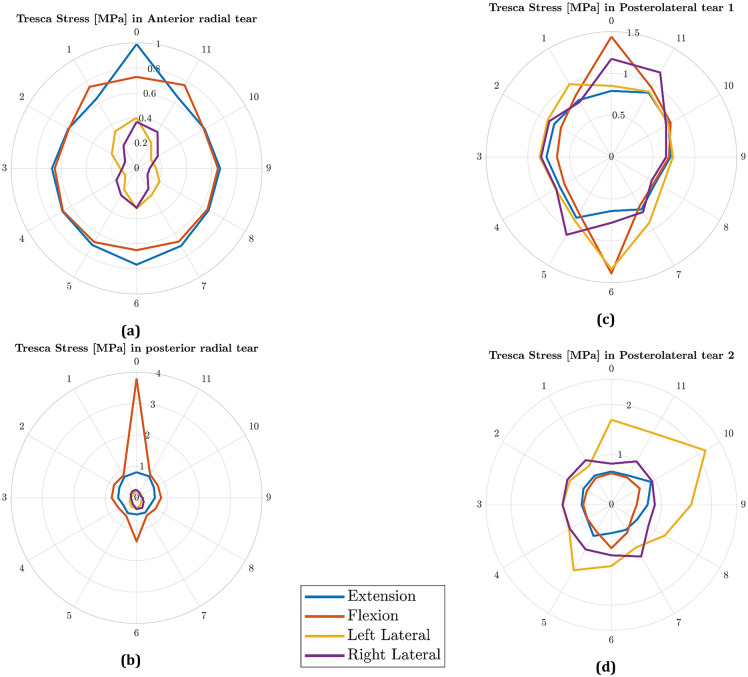
Polar plot for effects of movements on the boundary of (a) Anterior radial tear, (b) Posterior radial tear, (c) Posterolateral tear 1, (d) Posterolateral tear 2, in moderately degenerated annulusannulus.

Figs 25–27 show the effect of the four physiological movements on Tresca stress variations in the tear boundary for healthy, mild and moderate levels of material degeneration levels, respectively, for the four tear locations. Across all degeneration states, the sensitivity of each radial tear location to specific physiological movements follows a distinct and consistent pattern. Anterior tears show their highest stress under extension, with boundary stresses roughly double those observed in lateral bending and significantly higher than in flexion, indicating that extension is the primary aggravating motion regardless of degeneration. Posterior tears, in contrast, are dominated by flexion, where the peak stress increases 4–6-fold compared to all other motions, making flexion clearly hazardous movement for these tears. Both posterolateral tears exhibit strong susceptibility to lateral bending, but with different directional preferences: PL1 is consistently most stressed during left lateral bending (about 2x higher than other movements), while PL2 shows a characteristic shift, being driven primarily by right lateral bending in the healthy state, but transitioning to left lateral bending as the dominant stress‑inducing motion in mild and moderate degeneration, with 3-5x higher stresses than extension or flexion. Overall, these plots confirm that movement type interacts strongly with tear location, and degeneration amplifies stress magnitudes without altering the fundamental motion‑specific vulnerability patterns, except for the directional shift observed in PL2.

### Effect of degeneration on range of motion (ROM)

The effect of degeneration on ROM was observed regardless of the type and location of tears. The results were the same as those of our previous work on anterior circumferential tears [11]. The ROM decreased for all the movements considered with degeneration due to the increased stiffness. This resulted in an unusual decrease in the maximum Tresca stress for lateral movements.

### Comparison of results from contour plots and polar plots

**Fig 28:**
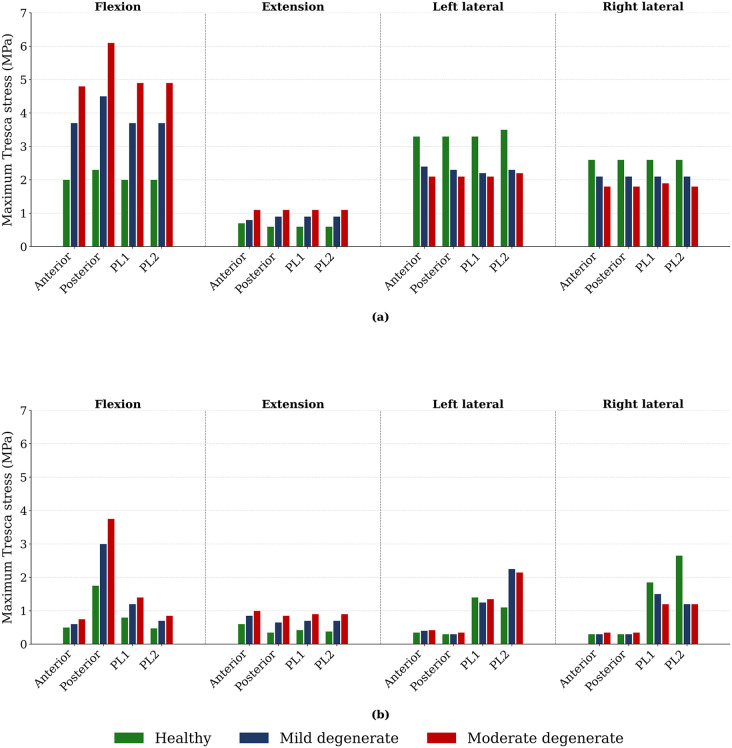
Bar graphs with maximum Tresca stress in (a) the mid-plane of the annulus and (b) the boundary of the tear.

The bar graphs in Fig 28 exhibit the trends of the maximum Tresca stress at the mid-plane and at the tear boundary for different movements across all tear locations and degeneration states. Under flexion, the mid-plane stress substantially increased with advancing degeneration across all tear locations. The mid-plane with posterior tear demonstrated the most pronounced change in stress from 2.3 MPa in the healthy state to 6.1 MPa in the moderately degenerated state, representing an increase of approximately 165%. The anterior, PL1, and PL2 tears produced comparable mid-plane stress values of approximately 2.0 MPa, 3.7 MPa, and 4.9 MPa across the three degeneration states respectively, reflecting a similarly significant increase of approximately 145%. At the tear boundary under flexion, the posterior tear exhibited the most critical stress concentration, increasing from 1.75 MPa to 3.75 MPa with advancing degeneration, followed by PL1 with values varying from 0.8 MPa to 1.4 MPa, while the remaining tear locations recorded comparatively lower boundary stress values ranging from 0.5 MPa to 0.85 MPa.

Under extension, both the mid-plane and tear boundary stresses increased modestly with degeneration and remained broadly comparable across all tear locations. This uniformity across tear locations confirms that tear position exerts negligible influence on the stress state under extension loading.

Under both lateral bending directions, the mid-plane stress demonstrated a reversal of the trend observed under flexion and extension. Under left lateral bending, the mid-plane stress decreased from approximately 3.2 to 3.5 MPa in the healthy state to approximately 2.1 MPa in the moderately degenerated state, representing a reduction of approximately 35–40%. Under right lateral bending, a comparable reduction was observed, with mid-plane stress decreasing from approximately 2.6 MPa in the healthy state to approximately 1.8 MPa in the moderately degenerated state, corresponding to a reduction of approximately 30%. This consistent reduction across both lateral bending directions is attributed to the restriction of segmental motion caused by annular stiffening induced by degeneration.

A particularly critical finding emerges from the tear boundary stress under lateral bending. While the mid-plane stress was broadly independent of tear location under both lateral bending directions, the tear boundary stress revealed a strikingly location-specific response, with PL1 and PL2 tears exhibiting substantially elevated boundary stresses of up to 2.2 MPa under left lateral bending and up to 2.65 MPa under right lateral bending, exceeding the corresponding stresses at the anterior and posterior tear boundaries by a factor of six to seven under the same loading conditions. This divergence between mid-plane and boundary stress responses demonstrates that mid-plane stress alone is insufficient to capture the full mechanical vulnerability of the annulus, and that tear boundary stress analysis provides a considerably more sensitive and discriminating indicator of tear-specific mechanical risk. Collectively, these findings identify the posterolateral tears as the most mechanically vulnerable configuration across multiple loading conditions, with their susceptibility persisting across all degeneration states examined.

## Discussions

### Clinical relevance of the results

The mechanical findings of this study offer biomechanical insight into how tear location and tissue degeneration interact with spinal loading directions, and may serve as a basis for generating hypotheses in future investigative work. The state of high stress around the tears and the tendency of the tears to open were considered supportive factors for the growth of tears. Similar to previous studies [20–22], we observe that degeneration and tears strongly influence the mechanical environment of the annulus. However, our study goes beyond these by simultaneously incorporating both radial tears and annular material degeneration — an aspect not addressed in prior finite element investigations. The stiffening of the annulus caused by degeneration contributed to stress concentration and impaired the ability of the disc to distribute load, thereby promoting further tear propagation and compromising disc function. The position of tears had a significant impact on depicting their criticality for various motions. The posterior and posterolateral tears tended to open during flexion. These findings were consistent with general clinical observations [8,42,43] and are now supported by a computational study combining degeneration effects and tear. These observations suggest that flexion may represent a mechanically unfavorable loading condition for individuals with posterior and posterolateral radial tears, a preliminary observation requiring further validation.

The extension movement facilitated closing of tears in the healthy annulus model, promoting a mechanically favorable environment for healing, which aligns with clinical observations [44]. However, in the degenerated models, extension was associated with elevated stress in the posterior and posterolateral region. While the absence of normative reference data prevents definitive conclusions regarding the clinical significance of this increase, the observed trend suggests that the mechanical environment during extension may become progressively less favorable with advancing degeneration, and demands further investigation. The anterior tear tended to open only during extension motion, accompanied by critical stress concentration at the tear tip towards the nucleus. Tear tips located closer to the nucleus are particularly vulnerable due to poor vascularity, which limits healing. This condition poses a risk of tear extension into the nucleus pulposus, potentially allowing nuclear material to reach the innervated annulus and cause pain [45]. While tear tips near the periphery have better healing potential due to relatively greater blood supply and innervation, repeated loading may still hinder recovery [46–49]. These observations suggest that extension may impose a mechanically unfavorable loading environment on anterior tears, whereas flexion, which promoted tear closure in the present study, may offer comparatively more favorable conditions for healing. Nevertheless, these findings are preliminary in nature and require further validation before they can inform any management strategies.

Lateral bending did not affect anterior and posterior tears but was associated with elevated stress in posterolateral tears in both bending directions. The results for all movements consistently present posterolateral tears as inherently susceptible to mechanical damage across all degeneration states, suggesting that this tear configuration may represent a particularly challenging pathological scenario. These mechanistic trends may offer a basis for future hypothesis generation regarding conservative management approaches, though they do not constitute clinical recommendations in the absence of comprehensive validation. The mechanical behaviours reported here provide biomechanical insight into how tear location and degeneration interact with physiological movements, offering preliminary indications that may inform future hypothesis-generation for conservative management strategies. These findings should not be interpreted as clinical recommendations, but rather as mechanistic trends that warrant further investigation using comprehensive verification, validation, and uncertainty quantification (VVUQ) frameworks before any clinical translation.

### Limitations and future scope

The present study establishes a controlled and rigorous comparative framework, and the following considerations outline the scope of this work and the directions along which it can be meaningfully extended. These include the use of a single subject-specific finite element model, the exclusion of time-dependent material behavior and complex physiological load combinations, and the absence of absolute clinical thresholds for tear progression. A single validated model, while appropriate for the comparative objectives of the present study, eliminates inter-specimen variability and ensures differences are unambiguously attributable to the variables of interest that precludes formal statistical testing and limits generalizability. Similarly, the adoption of pure moment loading, though deliberately chosen for its ability to produce uniform and unconfounded bending conditions suited to controlled comparative analysis, does not capture the full complexity of in vivo spinal mechanics, including compressive preloads, shear forces, and coupled multi-axial loading. Future investigations should address these limitations by expanding to a multi-specimen cohort, incorporating poroelastic and viscoelastic material behavior [50–54], and employing more physiologically comprehensive loading strategies such as follower load protocols or musculoskeletal model-derived boundary conditions.

Furthermore, the study identifies vulnerability patterns specific to each movement direction, tear location, and degeneration state, but does not define absolute clinical thresholds, such as specific flexion or extension angles, beyond which tear progression may worsen. The establishment of such thresholds requires experimental data quantifying the precise relationship between applied moments, angular kinematics, and tissue failure, which is currently unavailable in the literature. In the absence of such data, deriving clinically actionable limits from the present computational findings would be speculative. The findings should therefore be interpreted as relative, mechanistic trends that provide a foundation for future hypothesis generation, with the establishment of clinical thresholds recommended as an important direction for subsequent experimental and computational research.

## Conclusion

This exploratory and comparative computational study investigates the effect of radial tear location and annular tissue degeneration implemented through progressive stiffening of the annulus fibrosus, representing mildly and moderately degenerated states on the mechanical behaviour of the intervertebral disc under flexion, extension, and left and right lateral bending. Degeneration-induced stiffening of the annulus fibrosus was observed to produce a dominating effect on stress concentration and tear growth tendency, with its influence becoming increasingly pronounced with advancing degeneration severity. The posterior and anterior tears were observed to become mechanically critical during flexion and extension, respectively, while demonstrating relatively favourable mechanical conditions in the opposing movements which is a mechanistic trend that may develop future hypothesis regarding conservative management strategies, though it does not constitute a clinical recommendation in the absence of further experimental validation. However, the posterolateral tears consistently presented a tendency for damage progression across all four loading directions and degeneration states, suggesting that this tear configuration represents a particularly challenging mechanical scenario that may necessitate the consideration of alternative treatment strategies. Notably, stress state analysis near the tear boundary was found to be a more sensitive indicator of tear growth tendency than mid-plane stress analysis. These findings, interpreted as relative mechanistic trends within the defined scope of this comparative study, may assist in guiding future investigative frameworks aimed at understanding the relationship between tear location, tissue degeneration, and spinal loading.

## Supporting information

S1 File Polar plot data. (ZIP)
