# Supplementary material for: Progression of radial tears in L5-S1 intervertebral disc depends on location and type of movements: An in-silico study
Source: PLoS One. 2026 Jun 30;21(6):e0352680. doi: 10.1371/journal.pone.0352680 (PMC13318002; doi:10.1371/journal.pone.0352680)
Supplement: Attachment — Submitted filename: Response to Reviewers_R1.docx [file pone.0352680.s003.docx]

**Reviewer #1:**

The authors present a well-organized and relevant finite element study examining the mechanical effects of degeneration and radial tears in the L5–S1 disc under physiological motions. The modeling approach, incorporating anisotropic hyper-elastic behavior and graded degeneration, is thoughtfully executed and provides valuable biomechanical insights. Overall, this is a solid contribution. A few points, however, warrant further clarification and modifications to the manuscript. Please find my comments below.

Abstract:
1. Qualitative statements were while describing the results of the study. However, the readers would benefit if the authors included quantitative stats (% increase in stress, ROMs etc)

***Authors’ response:*** We thank the reviewer for this helpful suggestion. In response, we have revised the Abstract to incorporate quantitative comparisons derived from our polar‑plot and mid‑plane stress analyses. These include relative (x‑fold) increases in Tresca stress for different tear locations under specific physiological motions, for example, posterior tears exhibiting approximately 4-6x higher stresses during flexion, anterior tears showing 1.5-2x higher stresses during extension, and posterolateral tears demonstrating 2-5x higher stresses during lateral bending, depending on the tear subtype and degeneration level.

In addition to updating the Abstract, we have revised the corresponding sections in the Results and Discussion to expand the quantitative interpretation of:

- movement–location interactions,
- the effect of degeneration on stress amplification, and
- relative susceptibility of each tear location to specific motions.

These revisions enhance clarity and improve the scientific precision of the manuscript while aligning with the reviewer’s recommendation. The revised portions are highlighted in yellow in the revised manuscript.

Introduction:
1. [3] – full stop after reference

Ans – Thank you very much for this helpful observation. This suggestion is implemented in the draft.

2. “Many attempts have been made in the past to evaluate the effects of degeneration of the disc [13], but their focus was on the region above L5 vertebra, and generic models were used [13], [16].” – This statement is not accurate, and many studies modeled these effects before, with more levels included. I advise the authors to conduct a comprehensive review of literature, to include more studies here.

***Authors’ response:*** We sincerely thank the reviewer for this thoughtful observation. We would like to clarify that the cited reference [13] is a review article authored by us, which summarized existing finite‑element (FE) models and highlighted that a substantial proportion of earlier studies used generic (non‑subject‑specific) disc/segment representations and focused on levels above L5. Our sentence sought to reflect those review findings, rather than claim exclusivity.

In response to the reviewer’s suggestion, we have revisited recent literature and expanded the Introduction to include representative multi‑level and level‑specific FE investigations of degeneration and surgical scenarios (e.g., L1–S1 or L5–S1 transforaminal lumbar interbody fusion models, etc.), thereby providing a more balanced context. These additions now appear in the Introduction section of revised manuscript. The revised portions are highlighted in yellow in the revised manuscript.

We are grateful for the reviewer’s constructive feedback, which has helped enhance the completeness and clarity of the manuscript.

Methods:
1. Validation of the intact model – Although this has been discussed elsewhere, a brief discussion on what parameters were validated, and up to what extent will benefit this section or perhaps in the appendix if no of pages is a concern. This would set the stage for the intact model’s context of use in this study. This way, the present study is a standalone work with full context to the reader.

***Authors’ response:*** We thank the reviewer for highlighting the importance of model validation. The intact L5–S1 finite element model used in the present study has undergone exhaustive validation in our previously published work, wherein segmental range of motion under sagittal and coronal plane loading (flexion–extension and lateral bending) was systematically evaluated and shown to be in good agreement with experimentally reported data.

The validation procedure followed standard and widely accepted practices in spinal finite element analyses, where range of motion under physiological loading is considered a primary benchmark for model credibility. As this validation methodology is common across spinal FE analysis studies and has already been comprehensively documented, we have intentionally avoided repeating the same datasets and figures in the present manuscript to prevent redundancy and maintain focus on the novel aspects of degeneration and tear mechanics.

We believe that referencing the prior validation work provides sufficient context for the intact model’s credibility and intended use in this study. Reiterating the full validation results within the current manuscript or appendix would largely duplicate previously reported material without adding new insight. The validated intact model thus serves as a reliable baseline for the comparative analyses presented here.

2. Optionally, as a community practise, I would recommend consulting the ASME V&V 40 standard and the 2023 FDA guidance document on credibility assessment to establish model risk and credibility before its application (such as this study), since the conclusions of the study seem to be clinical in nature.

***Authors’*** *response:* We sincerely thank the reviewer for this insightful recommendation. We fully acknowledge the importance of the ASME V&V 40 standard and the 2023 FDA guidance on model credibility assessment, particularly for computational models intended to inform biomedical regulatory decisions, device evaluation, or high‑risk clinical applications.

In the present study, however, the FE model is employed primarily for exploratory biomechanical investigation to compare the relative effects of annular degeneration and tear location on stress distributions under different physiological movements. The model is not positioned as a tool/device for clinical decision‑supports, nor is it used to make regulatory claims. Therefore, a full ASME V&V 40–style risk‑based credibility assessment falls outside the intended scope and regulatory context of this work.

Nonetheless, we genuinely appreciate the reviewer’s perspective. We agree that as our future work would evolve toward higher‑risk applications, multi‑subject modeling, or clinically actionable predictions, integrating the ASME V&V 40 framework and FDA credibility recommendations will be both valuable and appropriate.

We thank the reviewer again for this constructive suggestion.

3. Material model – Does the model account for hysteresis and viscoelasticity? It would be interesting to see if the model can simulate progression of tears with repeated cyclic loading as observed in practise.

***Authors’ response:*** Thank you for the suggestion. The model does not include hysteresis or viscoelasticity effects. Since the simulation was static in nature, the time dependent properties were neglected. The inclusion of such properties is planned for the future and is mentioned in future studies. This has been highlighted in the Limitations section.

4. PL1 and PL2 in Figure 3 – I can not find the definition of these models in the methods section. What is the difference between these two? Geometry of the tears?

***Authors’ response:*** Thank you for the query. PL1 and PL2 are acronyms which stand for **P**ostero**L**ateral tear **1** and **P**ostero**L**ateral tear **2** and it is mentioned in the caption of Figure 3. These tears vary in terms of the relative position in the annulus as shown in Figure 3.

5. Material model of degeneration – ITM and k2 parameters. As I mentioned above, even if they are published elsewhere, context of these parameters is essential to understand how they influence the model. Is there any past evidence that the degenerated discs exhibit higher stiffness at larger strains?

***Authors’ response:*** We sincerely thank the reviewer for raising this important point regarding the role of the ITM and *k₂* parameters in representing degeneration within the GOH material framework. In our study, the degeneration‑related modifications to the annulus material were not arbitrarily chosen but are based directly on experimental evidence and on a previously published, peer‑reviewed modelling framework developed by us [12]. In that work, we demonstrated that annulus fibrosus stiffness increases with degeneration across the entire strain regime, consistent with multiple studies reporting elevated modulus in degenerated tissue. Specifically, Natarajan et al. [3] and Malandrino et al. [31] reported up to a 4.7‑fold increase in Young’s modulus of the annulus ground matrix in severely degenerated discs, confirming that degenerated discs exhibit higher stiffness not only at small strains but also at large strains under multiaxial loading.

To incorporate this physiologically supported stiffening into the GOH framework, our previous work derived the initial tangent modulus (ITM) analytically via Taylor‑series expansion of the constitutive model under equibiaxial stretch. This allowed us to adjust the small‑strain stiffness independently of *k₂*. Conversely, *k₂* was shown to be the primary parameter governing high‑strain nonlinearity, enabling its controlled modification to reproduce the experimentally observed stiffening at larger strains. This combined ITM–*k₂* approach produced stress–strain curves consistent with the experimental biaxial data of O’Connell et al. (2012) across the full strain range.

In the present manuscript, we adopt this same validated, literature‑supported approach. We have now clarified this context in the revised text as suggested by the reviewer.

The revised portions are highlighted in yellow in the revised manuscript.

We thank the reviewer for prompting this elaboration.

**Ref:** O’Connell G D, Sen S and Elliott D M. 2012. Human annulus fibrosus material properties from biaxial testing and constitutive modeling are altered with degeneration Biomech. Model. Mechanobiol. 11 493–503

6. Table 1 – Nucleus [29] [12]. Can the authors clarify what parameters were taken from [29]? This study does not talk about degeneration models. Is there a reason for the choice of 2MPa and 3MPa for mild and moderate cases?

***Authors’ response:*** We thank the reviewer for this helpful query regarding the selection of nucleus material properties in Table 1. Reference [29] was used specifically for the baseline material properties of a *healthy* nucleus pulposus, as it provides the elastic modulus and Poisson’s ratio values commonly adopted in validated spine FE models. While [29] does not include degeneration modelling, it serves as an established source for intact nucleus properties.

With degeneration, however, several studies [3] including our own previous in‑silico work [12] have reported that the nucleus pulposus begins to exhibit mechanical behaviour more similar to the annulus ground matrix, primarily due to biochemical changes such as reduced proteoglycan content, increased fibrosis, and higher solid‑phase stiffness. In our earlier published model [12], we therefore increased the stiffness of the nucleus proportionately to the increase applied to the annulus.

Following this same validated approach, the current study assigns 2 MPa and 3 MPa as Young’s moduli for mild and moderate degeneration, respectively. These values are proportional increases from the healthy value reported in [29] and match the modelling strategy adopted in our prior work [12], where we also simulated the degenerated nucleus by increasing its modulus to reflect its progressively more fibrotic, annulus‑like behaviour.

We have now clarified this reasoning in the revised manuscript to avoid ambiguity. The revised portions are highlighted in yellow in the revised manuscript.

7. 4Nm load – Is there a reason why the authors did not opt for the standard 7.5Nm load?
Perhaps a brief explanation in many of the above points would largely alleviate my concerns.

*Authors’ response:* We thank the reviewer for this important observation. The choice of 4 Nm was intentional and based on the mechanical behaviour of the annulus fibrosus in the non‑linear strain regime, which was the primary focus of this study. As explained in our previously published baseline model (Vinyas et al., 2023) [14], the annulus exhibits a markedly non‑linear response at lower applied moments, particularly in the region up to approximately 4 Nm. Beyond this magnitude, the stress–strain curve begins to transition toward a more linear behaviour, where incremental changes in moment produce comparatively linear changes in rotation and stress. Because the objective of this study is to analyse the behaviour of radial tears and degeneration under conditions where the annulus is most sensitive and mechanically unstable, it was essential to remain within the non‑linear regime where material stiffening, fibre recruitment, and tear‑opening behaviour are most pronounced.

Using 7.5 Nm, although commonly adopted for range‑of‑motion validation, would have placed the model closer to the linear region of annulus behaviour, thereby reducing sensitivity to the local mechanical effects of tear opening, tear‑tip stress amplification, and degeneration‑related stiffening. In contrast, 4 Nm provides a more physiologically relevant loading regime for analysing these subtle but clinically meaningful mechanical effects. This rationale is consistent with our validated work in [14], where the same load level was used to capture non‑linear annular behaviour.

We have now clarified this rationale in the revised manuscript. The revised portions are highlighted in yellow in the revised manuscript.

Results:
1. “information about the location of stress concentration can assist in identifying the site of discogenic pain [31].” overstates the evidence. To my knowledge, pain is a biopsychosocial phenomenon and cannot be inferred solely from stress maps produced by finite-element models. I recommend the authors modify this statement and cite more recent critical literature.

***Authors’ response:*** We thank the reviewer for this important and thoughtful comment. We fully agree that pain is a complex biopsychosocial phenomenon and cannot be directly inferred from finite element–derived stress distributions alone. The intent of the statement was not to suggest a direct or exclusive prediction of pain based on stress maps, but rather to highlight the biomechanical relevance of stress concentration as one of the contributing factors associated with discogenic pain, as discussed in the cited literature.

In the referenced study [31], stress concentration was identified as a mechanical criterion that may assist in understanding regions of increased vulnerability within the disc, particularly in the presence of degeneration or annular disruption, where nociceptive innervation is known to be present. Our statement was intended to be interpreted within this mechanistic context, rather than as a direct clinical diagnostic tool for pain.

To avoid overstatement and improve clarity, we have revised the sentence to explicitly acknowledge the multifactorial nature of pain and to frame stress concentration as a **supportive biomechanical indicator** rather than a determinant of discogenic pain. Where appropriate, we have also strengthened the discussion to emphasize that the findings provide biomechanical insight that may complement, but not replace, clinical assessment.

Here is the revised version for your reference.

“The information on the location of stress concentration may provide biomechanical insight into regions of increased structural vulnerability within the intervertebral disc, which can complement clinical understanding of discogenic pain, without implying a direct or sole prediction of pain based on mechanical measures alone [39].”

The revised portion is highlighted in yellow in the revised manuscript.

2. Use of Tresca criterion – this justification could be moved to the methods section.

***Authors’ response:*** Thank you for your valuable suggestion. Accordingly, we have moved this portion under the Methods section with sub-heading, “Shear stress based Tresca evaluation criterion”. The revised portions are highlighted in yellow in the revised manuscript.

3. Figure 10, 13, 16 – X axis label and Legend is missing. Data is incomplete.

***Authors’ response:*** Thank you for your kind observation. We apologize for the error in placing the graphs. These figures have now been updated in the revised manuscript.

4. It would also be interesting to see the trends in axial rotation (AR).

***Authors’ response:*** We thank the reviewer for this insightful suggestion. Axial rotation is indeed a relevant mode of spinal motion. In the present work, the analysis was conducted within the framework of a previously validated intact L5–S1 finite element model, for which exhaustive validation was performed under sagittal and coronal plane loading conditions. To maintain methodological consistency with this validated framework, the current study focused on these motion modes.

The trends observed under flexion–extension and lateral bending provide valuable biomechanical insight into the influence of degeneration and tear location. Extending this investigation to include axial rotation would be a natural and valuable next step, particularly following dedicated validation under torsional loading. We consider this an important direction for future studies building upon the present findings.

5. Polar plots – These plots are particularly interesting to see. However, it is hard to differentiate between mild and moderate. Perhaps use a different color.

***Authors’ response:*** We thank the reviewer for this important suggestion to improve readability of the figures. Accordingly, we have now updated Figures 17-21 with revised color coding for mild and moderate degeneration scenarios (blue and red, respectively).

6. Figure 18, 19, 20 – there is a mismatch between the sub plot titles a, b and the figure title. Flexion spelling in the sub plot.

***Authors’ response:*** We thank the reviewer for your keen observation. We have now corrected this mismatch of sub-plot figures and figure caption in the revised manuscript.

7. “Stress relaxing effect” – I suggest the authors use another word to describe the notion of insignificant increases in stress magnitude as degeneration progresses throughout the manuscript. Because “stress relaxation” is a viscoelastic phenomenon and not to be misinterpreted in the context of this study.

***Authors’ response:*** We thank the reviewer for this important clarification. We agree that the term “stress relaxation” has a specific meaning in the context of viscoelastic material behavior and is not appropriate for describing the observations in the present study. The intent was to convey that stress magnitudes exhibit a reduced response with progressive degeneration, rather than a time-dependent relaxation effect.

Accordingly, the term “stress relaxing effect” has been replaced in the manuscript with “reduction in stress magnitude with increasing degeneration.” We believe that this revision avoids potential misinterpretation. The revised portion is highlighted in yellow in the revised manuscript.

8. Effect of location of tears – “The first major observation here is the striking rise in stress by 2 to 3 times in the case of posterior tears compared to other tears under all conditions of annulus degenerations.” – I can not follow this statement as the plots show the rise only in flexion loading.

***Authors’ response:*** We thank the reviewer for pointing out this inconsistency. The statement was intended to refer specifically to flexion loading conditions. We acknowledge that this was not clearly stated in the original text. The sentence has now been revised to explicitly indicate that the observed 2–3-fold increase in stress for posterior tears occurs under flexion loading. This correction ensures consistency between the textual description and the plotted results. The revised portions are highlighted in yellow in the revised manuscript.

9. ROM changes – It would be interesting to see how the moment-rotation plots vary with degeneration and tear location. Is there any effect on neutral and extension zones due to these model changes? Perhaps the authors can include these plots in the appendix if not in the main body.

***Authors’ response:*** We thank the reviewer for this insightful comment. The effects of degeneration and annular tear location on moment–rotation behavior, including changes in neutral and extension zones, have been comprehensively investigated and reported in our previously published work titled *“In-silico study on cumulative effects of degeneration and anterior circumferential annular tear on the L5–S1 spinal unit.”* In that study, detailed moment–rotation plots and associated analyses were presented to elucidate the influence of degenerative changes on spinal kinematics.

To avoid duplication of previously published results and maintain focus on the novel aspects of the present study—namely, stress and strain alterations associated with different tear locations—we have not reproduced those plots here or in the appendix. Interested readers are referred to the earlier publication for an in-depth discussion of ROM-related changes. We believe this approach ensures clarity while preventing redundancy across studies.

10. Did the authors consider conducting any statistical tests to quantify the statistical significance of degeneration and tear location? This is highly recommended.

***Authors’ response:*** We thank the reviewer for this thoughtful suggestion. Statistical testing is indeed valuable in studies involving multiple subjects or experimental repetitions. However, the present investigation is a deterministic finite element–based numerical study conducted on a validated representative L5–S1 spinal unit. The objective of the study was to evaluate relative changes in biomechanical responses due to degeneration and tear location under controlled loading conditions, rather than to assess population-level variability.

As the simulations were performed on a single baseline model with systematically introduced degenerative conditions and tear configurations, conventional statistical analyses (e.g., hypothesis testing or significance measures) are not directly applicable. The observed differences in mechanical responses arise from controlled parametric variations within the same model framework and therefore represent deterministic outcomes rather than stochastic data.

We have clarified this aspect by emphasizing the comparative and mechanistic nature of the analysis. Future extensions of this work involving subject-specific models or multiple anatomical datasets would be more suitable for statistical evaluation. This is mentioned in the Limitations section of the manuscript. The revised portions are highlighted in yellow in the revised manuscript.

Discussion:
1. Clinical implications – ‘Flexion has increased risk of posterior tears; extension – anterior tears; lateral bending – lateral tears’ These qualitative inferences can be directly made solely by studying the geometry of tears from scans. But, after this numerical study, can any quantitative inferences be made? Such as, flexion above x degrees may be deteriorating in case of posterior tears etc.

***Authors’ response:*** We thank the reviewer for this insightful observation. We agree that establishing quantitative clinical thresholds such as, identifying specific flexion or extension angles at which tear progression becomes likely, would be highly valuable for translational application. However, the present study was not designed to derive such motion‑specific angular limits. Because our computational framework is based on a single subject‑specific spinal unit, and the applied pure‑moment loading does not map uniquely to clinical motion angles across individuals, estimating population‑relevant thresholds (e.g., “flexion above *x* degrees is harmful”) would extend beyond the intended use and credibility of the model.

Our objective was instead to perform a comparative biomechanical analysis to understand *how* tear location interacts with degeneration and movement type, rather than to define absolute kinematic tolerances. Within this scope, the current manuscript demonstrates quantitative, location‑specific approximate stress multipliers (e.g., posterior tears experiencing 4-6x higher stresses in flexion, anterior tears showing 1.5-2x higher stresses in extension, and PL2 tears showing 3-5x higher stresses in lateral bending). These quantitative comparisons are now explicitly incorporated into the revised Results and Abstract sections strengthen the mechanistic understanding of tear‑specific vulnerability patterns.

We fully agree that determining clinically relevant angular thresholds would require future work involving:

- multiple subject‑specific models to capture inter‑individual variability,
- mapping applied FE moments to subject‑specific angular ROM, and
- validation against in vivo kinematic data.

Thus, while our findings provide quantitative biomechanical evidence of movement‑specific susceptibility, defining absolute clinical cutoff angles was outside the scope of this study. We have clarified this in the Limitations section of the revised manuscript. The revised portions are highlighted in yellow in the revised manuscript.

2. “The mechanical behaviours reported here offer practical guidance on physical activity tailored for different age groups and tear conditions to mitigate damage and promote disc healing, thereby linking biomechanical insights with potential clinical strategies.” – This is an overstatement considering the above raised points and mentioned limitations of the study. Arguably, a much more comprehensive VVUQ study is needed to use this model as evidence in any clinical setting. I suggest the authors to rework these claims.

***Authors’ response:*** We thank the reviewer for pointing out the risk of overstating the clinical implications of our findings. We agree entirely that the current model being exploratory, single‑subject, and not supported by a full VVUQ framework, cannot be used to guide physical activity recommendations or clinical decision‑making. In response, we have carefully revised the relevant sentence in the Discussion section (under “Clinical relevance of the results” sub-section) to remove any implication of direct clinical applicability. The revised text now emphasizes that the results provide mechanistic, biomechanical insight rather than clinical guidance, and that any clinical translation would require a comprehensive VVUQ process, consistent with ASME V&V‑40 and FDA credibility expectations. This modification ensures our claims remain aligned with the scope and limitations of the present study.

The revised portions are highlighted in yellow in the revised manuscript.

3. 75% tear length – Are the model results sensitive to this parameter? A graph indicating progression of stress/ROM with % length of tear may be included. Without this discussion, the choice of 75% seems arbitrary without any clinical reasoning.

***Authors’ response:*** “Though not based on a specific clinical threshold, the length was intentionally chosen to reflect substantial structural disruption and to illustrate the biomechanical consequences of such tears.” – This reason is now included in the Method section. The revised portion is highlighted in yellow in the revised manuscript.

References:
1. Please check all the references to align with journal formatting requirements (e.g. 27, 30)

***Authors’ response:*** We thank the reviewer for highlighting the importance of aligning the reference list with the journal’s formatting requirements. In response, we have thoroughly reviewed and revised the entire reference list to ensure compliance with the format mandated by PLOS ONE. These updates have been applied uniformly throughout the manuscript, and the reference list now fully adheres to the journal’s style guidelines.

*We sincerely thank the reviewer for their careful reading, constructive feedback, and thoughtful recommendations throughout the review process. Their insights have significantly strengthened the scientific clarity, methodological transparency, and overall presentation of our manuscript. We truly appreciate the time and expertise invested in helping improve the quality of our work.*

**Reviewer #2:**

This manuscript presents a finite element study aimed at calculating peak stresses resulting from radial annulus fibrosus tears at different locations, under different modes of loading, and different stages of degeneration. While the effect of increased stress at the ends of the tear, particularly in states where the tissue is stiffest, is intuitive, the manuscript does contribute to the literature with its thoroughness. The text of the manuscript is clear, but the conclusions rely heavily on interpretation of figures, which are not always used effectively.

- Table 1 is missing and Figures 10, 13, and 16 are empty.

***Authors’ response:*** Thank you for your kind observation. We apologize for the error in placing the graphs. These figures have now been updated in the revised manuscript.

- Please indicate Right and Left when applicable.

***Authors’ response:*** Thank you for your valuable suggestion to improve understandability of the readers. Figure 3(a) has been updated to indicate the left and right directions of the annulus, respectively.

- Figure 8 shows an anterolateral stress peak that's on the order of the peak stresses around the tear. The reason for this should be clarified.

Geometrical undulations/shapes of the vertebra

***Authors’ response:*** We thank the reviewer for this important observation. The anterolateral stress peak observed in Figure 8 arises primarily from local geometric variations at the vertebral endplate–annulus interface. Subtle undulations and curvature changes in the endplate geometry can alter load transfer pathways, leading to localized stress amplification under physiological loading. In the applied loading configuration, the anterior annulus predominantly experiences tensile stresses, while the posterior region is subjected to compression. This asymmetric stress state, when combined with geometric nonuniformities, results in elevated anterolateral stress concentrations that are comparable in magnitude to those observed near the tear. These stress elevations are structural and geometry-driven in nature and should not be interpreted as tear-induced stress amplification.

This clarification is now included in the revised manuscript. The revised portion is highlighted in yellow in the revised manuscript.

- The text refers in multiple places to the intact model, but these results are not shown. While it is intuitive that a tear will increase stress at a particular location, the manuscript is unclear on the magnitude of that increase.

***Authors’ response:*** We thank the reviewer for this valuable observation. We agree that we refer to the intact model in several places. However, the primary focus of the present study is not to compare each tear configuration against the intact disc, but rather to analyse how tear *location* and degeneration level interact to influence the *relative* stress patterns within the annulus. The intact‑disc behaviour for the same subject‑specific model has already been extensively documented and validated in our previous work, which is cited in the manuscript. Because the present study is intended as a *comparative analysis among different tear locations*, we did not reproduce the intact model results again to avoid duplication.

That said, we appreciate that referring to the intact model may imply a quantitative comparison is expected. To address this, we have now:

1. Revised the phrasing in the Results section to avoid implying that a direct intact‑vs‑torn comparison is part of the present analysis.
2. Added explicit statements clarifying that the intact model results are available in our prior publication (cited in [14]) and that the present study is focused on *comparative trends among tear types and degeneration states* rather than absolute differences from intact.
3. Added brief quantitative descriptors in places where tear‑induced stress amplification is mentioned (e.g., “posterior tears experienced approximately 4-6x higher boundary stresses during flexion, while anterior tears showed 1.5-2x higher stresses during extension” in the Abstract and similar descriptions in the Results section), so the reader can appreciate the magnitude of changes within the tear‑location comparison framework.

These changes improve clarity while keeping the study aligned with its intended scope. The revised portions are highlighted in yellow in the revised manuscript.

- How do the peak Tresca stresses relate to ultimate strength of the AF tissue in each of the degeneration states?

***Authors’ response:*** We thank the reviewer for raising this important point. We fully agree that relating computed stresses to the ultimate mechanical strength of the annulus fibrosus (AF) would be valuable for interpreting tissue‑level failure risk. However, the present study focuses on comparative biomechanics (tear‑location x degeneration x movement) rather than on estimating absolute failure thresholds.

A direct comparison with ultimate strength was not performed for few key reasons:

1. Degeneration‑specific ultimate strength values for AF tissue are not available in the literature. Existing experimental studies report AF strength under isolated uniaxial or biaxial loading in healthy or pooled‑age samples, but they do not provide ultimate shear strength, nor stratification by mild vs. moderate degeneration.
2. Even commonly cited studies (e.g., O’Connell et al., 2012; Iatridis et al., 2004) provide modulus and nonlinear stress–strain behavior, but not failure stresses in shear, which is the mode relevant to Tresca stress.
3. Ultimate strength of AF tissue is highly dependent on test mode, hydration, fiber angle, and annular region, making direct stress‑to‑failure comparisons difficult without matched loading modes.
4. Published strength measures typically correspond to fiber‑direction tensile failure, which can exceed 3-6 MPa in healthy tissue but is not representative of shear‑driven delamination, where failure often initiates at much lower stress levels but is rarely quantified experimentally.

Given these limitations, we therefore interpret the Tresca stress fields comparatively (i.e., which tear locations and which movements amplify shear stresses the most), rather than as predictors of absolute failure. Our results identify relative susceptibility patterns, which are consistent with the stated aims of the present study.

We agree that integrating experimentally validated failure thresholds, especially under physiological shear and across degeneration grades, would be valuable and constitutes an important avenue for future work.

We have now clarified this aspect under the Methods section with sub-heading, “Shear stress based Tresca evaluation criterion”. The revised portions are highlighted in yellow in the revised manuscript.

*We sincerely thank the reviewer for their careful reading of our manuscript and for the constructive, insightful comments provided. Their observations have helped us substantially improve the clarity, scientific rigour, and overall quality of the work. We truly appreciate the time and effort invested in strengthening this study.*

**Ref:** O’Connell G D, Sen S and Elliott D M. 2012. Human annulus fibrosus material properties from biaxial testing and constitutive modeling are altered with degeneration Biomech. Model. Mechanobiol. 11 493–503
