# Supplementary material for: Progression of radial tears in L5-S1 intervertebral disc depends on location and type of movements: An in-silico study
Source: PLoS One. 2026 Jun 30;21(6):e0352680. doi: 10.1371/journal.pone.0352680 (PMC13318002; doi:10.1371/journal.pone.0352680)
Supplement: Attachment — Submitted filename: Response to Reviewers_R2.docx [file pone.0352680.s004.docx]

**Reviewer #2:** All of my comments from the prior review have been satisfactorily addressed in the revised manuscript.

***Response:*** Thank you for your valuable comments and suggestions, which have enriched the manuscript.

**Reviewer #3:**

GENERAL COMMENT

This paper presents on a computational study aimed to assess the effect of the presence and location of radial tears in the annulus fibrosus, and tissue quality, on the damage occurring to the annulus fibrosus under extension, flexion and bending moments.

The authors used a previously developed finite element model of the L5-S1 compartment, which was modified to simulate the effect of ageing on the material properties of the intervertebral disc. Three different versions of the model were thus generated, corresponding to a healthy, a mildly degenerated, and a moderately degenerated tissue.

For each model, different scenarios were tested by modifying the boundary conditions. Four different loading conditions were tested: pure flexion, pure extension and pure left/right lateral bending moment. Four different tear locations were simulated: anterior, posterior and posterolateral (PL1 and PL2). The output of the simulations was compared in terms of maximum Tresca stress.

The manuscript has significantly improved upon the first round of revisions, as the authors carefully addressed most of the reviewers’ comments. However, I have some concerns on how the results are presented and interpreted.

In light of the modelling choices (applied loads and material properties of the tissues) and the nature of the work that the authors themselves label as comparative and exploratory, I struggle to see any direct clinical implications of this work. I suggest the authors to revise the discussion section, by tuning down some statements, and to refrain from providing clinical suggestions based on the presented results.

Furthermore, to strengthen the work, it would be good to see some numbers accompanying the text in the results section. Although direct comparisons to clinical and/or normative healthy data may not be possible, reading the values would help appreciating the differences between tested conditions. Last, the quality of the figure could be improved to facilitate qualitative comparisons.

Please see my specific comments below.

SPECIFIC COMMENTS

- Methods –

1. Please clarify the link between the applied boundary conditions and real loading conditions. Are human intervertebral discs commonly experiencing those types of loads (pure moments) and to the same extent (4 Nm), during activities of daily living? In other words, how representative of in vivo loads are the implemented boundary conditions?

***Response:***

We thank the reviewer for this valuable comment. The application of **pure moments** is a widely adopted approach in experimental and computational studies of spinal segments because it enables the evaluation of the intrinsic mechanical response of the motion segment without introducing additional complexities associated with muscle forces and follower loads. The magnitude of **4 Nm** falls within the commonly reported range used in in vitro biomechanical experiments and finite element studies of lumbar motion segments, thereby facilitating meaningful comparisons with the existing literature.

While such loading conditions do not fully capture the in vivo loading during daily activities, they are considered a standardized, physiologically relevant benchmark for assessing the biomechanical behavior of spinal units during typical motions such as forward, backward, and lateral bending. Therefore, the implemented boundary conditions were selected to ensure consistency with established spinal biomechanics methodologies and to allow the results to be interpreted in the context of previously reported experimental and numerical studies. A brief clarification has been included in the manuscript to reflect this rationale.

2. “Boundary conditions were designed to simulate four physiological movements: flexion, extension, left lateral bending, and right lateral bending, in the presence of a compressive load. A bending moment of 4 Nm was applied to a central reference point on the superior endplate of the L5 vertebra in all simulations.”

COMMENT: Please clarify where and how the compressive load was applied.

***Response:***

We thank the reviewer for pointing out this ambiguity in our manuscript. We apologize for the confusion caused by the unclear description of the boundary conditions.

To clarify, **no separate compressive load was applied** in addition to the bending moment. The phrase "in the presence of a compressive load" was intended to describe the **inherent compressive component** that arises at the L5-S1 joint due to the application of the bending moment itself about the reference point on the superior endplate of L5, not an independently prescribed axial force.

We recognize that this phrasing was misleading and have revised the manuscript accordingly. The corrected sentence now reads:

*"* *Boundary conditions were designed to simulate four physiological movements: flexion, extension, left lateral bending, and right lateral bending. A bending moment of 4 Nm was applied to a central reference point on the superior endplate of the L5 vertebra in all simulations.”*

- Results -

3. Please add numbers/values (e.g. for the peak stresses) directly in the text of the results section to provide some quantitative assessment.

***Response***- We thank the reviewer for this important suggestion. In the revised manuscript, quantitative peak Tresca stress values (in MPa) have been added directly to the text throughout the Results section for each loading condition, tear location, and degeneration state. Specifically, numerical values have been inserted into the subsections on flexion, extension, left lateral bending, right lateral bending, the polar plot degeneration analysis, the tear location comparison, the movement effect analysis, and the contour-versus-polar-plot comparison. These additions provide the quantitative context requested, enabling direct numerical appreciation of the differences between tested conditions.

Discussion

4. As mentioned in the general comment, considering the nature of the work (exploratory and comparative, as acknowledged by the authors) and specific modelling choices, it is hard to see direct clinical implications of this study. Please revise the subsection ‘Clinical relevance of the results’, starting from the opening sentence, to reflect this.

***Response-*** We thank the reviewer for this valid and important observation. We fully agree that characterizing the findings as having direct clinical implications overstates the translational scope of a study that, by design, is exploratory and comparative. The opening sentence has been revised to more accurately reflect the scope of the work. Specifically, the phrase *"direct clinical and biological implications"* has been replaced with language that frames the findings as biomechanical insights that may serve as a basis for hypothesis generation in future investigations. Throughout the revised subsection, clinical language has been systematically replaced with appropriately hedged mechanistic framing, consistent with the exploratory nature of the study. We believe these revisions more accurately represent the contribution and scope of the present work.

5. “However, extension may be unsafe for older individuals, as degeneration increases the stress in the posterior and posterolateral region, which can further damage surrounding tissues and accelerate tear growth.”

COMMENT: In the absence of normative data to compare the reported results with, one cannot conclude whether the observed increase can be dangerous or not.

***Response***- We thank the reviewer for this technically precise and valid observation. We agree that, in the absence of normative reference data establishing thresholds for tissue damage or failure, it is not possible to conclude whether the observed stress increase in the posterior and posterolateral region during extension constitutes a genuinely dangerous condition. The original statement has been revised to remove this unsubstantiated conclusion. The revised text now acknowledges the elevated stress trend in degenerated models under extension while explicitly noting that the absence of normative data precludes definitive conclusions regarding its clinical significance. The finding is instead framed as a mechanistic trend warranting further investigation, consistent with the study's exploratory scope.

6. “Therefore, lateral bending motions should be strictly avoided for patients with posterolateral tears, regardless of age, which corroborates clinical findings [50].”
COMMENT: Please refrain from providing clinical indications. The presented results do not support such statements. For instance, it is not clear whether the applied moments – that differ from what is commonly used in similar computational studies - reflect any specific habitual conditions (e.g., activities of daily living or physiotherapy exercises). In addition, ref 50 does not seem appropriate.

***Response*** - We thank the reviewer for this advice. We agree that the statement *"lateral bending motions should be strictly avoided for patients with posterolateral tears, regardless of age"* constitutes a direct clinical recommendation that the present results do not adequately support. Specifically, as the reviewer correctly notes, the applied pure moments do not correspond to any precisely defined habitual activity or physiotherapy exercise, and therefore cannot serve as the basis for prescriptive clinical guidance. This statement has been removed in its entirety from the revised manuscript. The finding has been reframed to note that lateral bending was associated with elevated stress in posterolateral tears across all degeneration states, presenting this as an observation that may inform future hypothesis generation rather than a clinical directive. Additionally, reference [50] has been removed, as we agree with the reviewer's assessment that it is not appropriate in this context.

7. “The clinical translation of techniques such as annular sealants, still under development, offers promise for treating such injuries earlier and more effectively [51]–[53].”
COMMENT: This whole sentence appears out of context, and should be removed.

**Response** - We thank the reviewer for this observation and agree that the sentence regarding annular sealants was tangential to the mechanical findings presented and inconsistent with the exploratory scope of the study. The sentence has been removed in its entirety from the revised manuscript. The discussion now maintains focus on the mechanistic findings and their potential role in informing future investigative frameworks, without extending into broader therapeutic domains beyond the scope of the present work.

8. In the limitations section, please state each limitation first and then explain what could be done differently and why you did it the way you did.

**Response** - We thank the reviewer for this constructive suggestion. The limitations section has been comprehensively restructured in accordance with the recommended format. The revised section concisely enumerates all key limitations upfront, followed by a cohesive, integrated discussion that explains the rationale for the adopted approaches and the directions recommended for future work. The absence of clinical thresholds has been accurately attributed to the current unavailability of experimental data establishing the precise relationship between applied moments, angular kinematics, and tissue failure. We believe these revisions improve the transparency, completeness, and readability of the limitations section.

- Conclusion –

9. It is important to emphasize here that this is an exploratory and comparative study.

**Response-** We thank the reviewer for this important reminder. The opening sentence of the conclusion has been revised to explicitly characterize the study as exploratory and comparative in nature. This framing is now consistently maintained throughout the conclusion, ensuring that the findings are presented as mechanistic trends within a defined investigative scope rather than definitive or generalizable conclusions.

10. The term ageing was used only once before in text, in the introduction. It may not be clear to the readership what the authors are referring to here. Be consistent with the terminology and refrain from using keywords only in the introduction and conclusions sections, for sake of clarity. It may be worth, throughout the manuscript, to remind the readers that the effect of ageing was implemented by increasing the stiffness of the annulus fibrosus (i.e., mildly degenerated, and moderately degenerated states).

**Response-** We thank the reviewer for identifying this inconsistency. The term "ageing" has been removed from the conclusion and replaced with terminology that is consistent with that used throughout the manuscript — specifically, the progressive stiffening of the annulus fibrosus representing mildly and moderately degenerated states. Furthermore, we have revised the manuscript to include periodic reminders throughout the methods, results, and discussion sections that degeneration was implemented by increasing annular stiffness in mildly and moderately degenerated tissue states, ensuring terminological clarity and consistency for the readership.

11. “This indicated that the presence of these tears could essentially be healed by conservative treatments of physiotherapy.”

COMMENT: The presented data does not support any such statements.

**Response-** We thank the reviewer for this valid and important observation. We agree that the original statement — suggesting that posterior and anterior tears could be healed through conservative physiotherapy — constitutes an unsupported clinical recommendation that exceeds what the presented computational data can substantiate. This statement has been removed in its entirety. In its place, the revised conclusion acknowledges the mechanically favorable conditions observed in opposing movements as a mechanistic trend that may inform future hypothesis generation, while explicitly stating that this does not constitute a clinical recommendation in the absence of further experimental validation. This revision is consistent with the exploratory scope of the study and the broader revisions made to the discussion section.

- Figures -

12. Please ensure that the various subplots in the figures use the same scales (i.e., same y limits for the colormap), otherwise the pictorial representation and visual information can be misleading (this is valid for both contours and radar plots).

**Response** - We thank the reviewer for this important observation regarding the consistency of visual representations across subplots. We would like to respectfully clarify that the contour plots presented for each tear configuration already use a unified colormap across all three degeneration states — healthy, mild degeneration, and moderate degeneration — as shown in the accompanying figure. The minimum and maximum limits of the colormap are held constant across all subplots within a given tear configuration, ensuring that the color contours are directly comparable across degeneration states without any visual distortion or misleading representation.

Specifically, the colormap range has been set based on the maximum Tresca stress value observed across all degeneration conditions for a given tear location, so that the stress distributions across healthy, mildly degenerated, and moderately degenerated models are presented on an identical scale. This approach ensures that any apparent differences in color distribution across the subplots genuinely reflect differences in the underlying stress state rather than artifacts of inconsistent scaling.

We have carefully reviewed all contour figures and radar plots in the manuscript to ensure consistency throughout, and have made the necessary adjustments where inconsistencies were identified. We hope this clarification adequately addresses the reviewer's concern.

13. Figure 28 could be improved by grouping the results in different subplots (or at least by showing the two parts - a and b – above each other) and should be moved to the Results section. All the results (and figures) should be introduced before the discussion.

**Response** - We thank the reviewer for these constructive suggestions regarding the presentation and placement of Figure 28. We have addressed both aspects of this comment as follows.

Regarding the layout of Figure 28, we agree that the original arrangement of the two parts did not facilitate effective visual comparison. The figure has been revised such that parts (a) and (b) are now presented above each other in a vertically stacked arrangement, enabling direct side-by-side qualitative comparison between the two conditions. This reorganization improves the readability and interpretive clarity of the figure without altering the underlying data or results presented.

Regarding the placement of Figure 28, we agree with the reviewer's observation that all results and their corresponding figures should be presented in the Results section prior to the Discussion. This is a well-established convention in scientific reporting that ensures the reader has access to the complete set of findings before their interpretation is offered. Figure 28 has accordingly been relocated to the Results section, at the appropriate position consistent with the sequence in which the corresponding findings are reported. We have reviewed all remaining figures and results throughout the manuscript to ensure that this convention is consistently upheld, and no other figures were found to be misplaced in this regard.

We trust that these revisions adequately address the reviewer's concerns and improve both the visual presentation and structural organization of the manuscript.
