# Supplementary material for: Progression of radial tears in L5-S1 intervertebral disc depends on location and type of movements: An in-silico study
Source: PLoS One. 2026 Jun 30;21(6):e0352680. doi: 10.1371/journal.pone.0352680 (PMC13318002; doi:10.1371/journal.pone.0352680)
Supplement: Peer review history

# Peer review: Decision Letter 0

**DOI:** 10.1371/journal.pone.0352680.r001

**Authors:** Alessandra Aldieri

17 Nov 2025

PONE-D-25-46137

Progression of radial tears in L5-S1 intervertebral disc depends on location and type of movements: An in-silico study

PLOS ONE

Dear Dr. Bhat,

Thank you for submitting your manuscript to PLOS ONE. After careful consideration, we feel that it has merit but does not fully meet PLOS ONE’s publication criteria as it currently stands. Therefore, we invite you to submit a revised version of the manuscript that addresses the points raised during the review process.

The Reviewers have highlighted the value of the work, but some issues were pointed out, mainly formal.

Please submit your revised manuscript by Jan 01 2026 11:59PM. If you will need more time than this to complete your revisions, please reply to this message or contact the journal office at plosone@plos.org. When you're ready to submit your revision, log on to https://www.editorialmanager.com/pone/ and select the 'Submissions Needing Revision' folder to locate your manuscript file.

Please include the following items when submitting your revised manuscript:

- A rebuttal letter that responds to each point raised by the academic editor and reviewer(s). You should upload this letter as a separate file labeled 'Response to Reviewers'.
- A marked-up copy of your manuscript that highlights changes made to the original version. You should upload this as a separate file labeled 'Revised Manuscript with Track Changes'.
- An unmarked version of your revised paper without tracked changes. You should upload this as a separate file labeled 'Manuscript'.

If you would like to make changes to your financial disclosure, please include your updated statement in your cover letter. Guidelines for resubmitting your figure files are available below the reviewer comments at the end of this letter.

If applicable, we recommend that you deposit your laboratory protocols in protocols.io to enhance the reproducibility of your results. Protocols.io assigns your protocol its own identifier (DOI) so that it can be cited independently in the future. For instructions see: https://journals.plos.org/plosone/s/submission-guidelines#loc-laboratory-protocols. Additionally, PLOS ONE offers an option for publishing peer-reviewed Lab Protocol articles, which describe protocols hosted on protocols.io. Read more information on sharing protocols at https://plos.org/protocols?utm_medium=editorial-email&utm_source=authorletters&utm_campaign=protocols.

We look forward to receiving your revised manuscript.

Kind regards,

Alessandra Aldieri

Academic Editor

PLOS ONE

Journal Requirements:

When submitting your revision, we need you to address these additional requirements.

1. Please ensure that your manuscript meets PLOS ONE's style requirements, including those for file naming. The PLOS ONE style templates can be found at

https://journals.plos.org/plosone/s/file?id=wjVg/PLOSOne_formatting_sample_main_body.pdf and https://journals.plos.org/plosone/s/file?id=ba62/PLOSOne_formatting_sample_title_authors_affiliations.pdf

2. Please note that PLOS One has specific guidelines on code sharing for submissions in which author-generated code underpins the findings in the manuscript. In these cases, we expect all author-generated code to be made available without restrictions upon publication of the work. Please review our guidelines at https://journals.plos.org/plosone/s/materials-and-software-sharing#loc-sharing-code and ensure that your code is shared in a way that follows best practice and facilitates reproducibility and reuse.

3. In the online submission form, you indicated that [The obtained results are provided in detail in the manuscript. If further data is needed on modeling and simulation approaches, the corresponding author may be contacted for clarifications.].

All PLOS journals now require all data underlying the findings described in their manuscript to be freely available to other researchers, either 1. In a public repository, 2. Within the manuscript itself, or 3. Uploaded as supplementary information.

This policy applies to all data except where public deposition would breach compliance with the protocol approved by your research ethics board. If your data cannot be made publicly available for ethical or legal reasons (e.g., public availability would compromise patient privacy), please explain your reasons on resubmission and your exemption request will be escalated for approval.

If the reviewer comments include a recommendation to cite specific previously published works, please review and evaluate these publications to determine whether they are relevant and should be cited. There is no requirement to cite these works unless the editor has indicated otherwise.

[Note: HTML markup is below. Please do not edit.]

Reviewers' comments:

Reviewer's Responses to Questions

**Comments to the Author**

1. Is the manuscript technically sound, and do the data support the conclusions?

The manuscript must describe a technically sound piece of scientific research with data that supports the conclusions. Experiments must have been conducted rigorously, with appropriate controls, replication, and sample sizes. The conclusions must be drawn appropriately based on the data presented.

Reviewer #1: Yes

Reviewer #2: Partly

**********

2. Has the statistical analysis been performed appropriately and rigorously?

Reviewer #1: No

Reviewer #2: N/A

**********

3. Have the authors made all data underlying the findings in their manuscript fully available?

The PLOS Data policy requires authors to make all data underlying the findings described in their manuscript fully available without restriction, with rare exception (please refer to the Data Availability Statement in the manuscript PDF file). The data should be provided as part of the manuscript or its supporting information, or deposited to a public repository. For example, in addition to summary statistics, the data points behind means, medians and variance measures should be available. If there are restrictions on publicly sharing data—e.g. participant privacy or use of data from a third party—those must be specified.

Reviewer #1: Yes

Reviewer #2: Yes

**********

4. Is the manuscript presented in an intelligible fashion and written in standard English?

PLOS ONE does not copyedit accepted manuscripts, so the language in submitted articles must be clear, correct, and unambiguous. Any typographical or grammatical errors should be corrected at revision, so please note any specific errors here.

Reviewer #1: Yes

Reviewer #2: Yes

**********

5. Review Comments to the Author

Please use the space provided to explain your answers to the questions above. You may also include additional comments for the author, including concerns about dual publication, research ethics, or publication ethics. (Please upload your review as an attachment if it exceeds 20,000 characters)

Reviewer #1: The authors present a well-organized and relevant finite element study examining the mechanical effects of degeneration and radial tears in the L5–S1 disc under physiological motions. The modeling approach, incorporating anisotropic hyper-elastic behavior and graded degeneration, is thoughtfully executed and provides valuable biomechanical insights. Overall, this is a solid contribution. A few points, however, warrant further clarification and modifications to the manuscript. Please find my comments below.

Abstract:

1. Qualitative statements were while describing the results of the study. However, the readers would benefit if the authors included quantitative stats (% increase in stress, ROMs etc)

Introduction:

1. [3] – full stop after reference

2. “Many attempts have been made in the past to evaluate the effects of degeneration of the disc [13], but their focus was on the region above L5 vertebra, and generic models were used [13], [16].” – This statement is not accurate, and many studies modeled these effects before, with more levels included. I advise the authors to conduct a comprehensive review of literature, to include more studies here.

Methods:

1. Validation of the intact model – Although this has been discussed elsewhere, a brief discussion on what parameters were validated, and up to what extent will benefit this section or perhaps in the appendix if no of pages is a concern. This would set the stage for the intact model’s context of use in this study. This way, the present study is a standalone work with full context to the reader.

2. Optionally, as a community practise, I would recommend consulting the ASME V&V 40 standard and the 2023 FDA guidance document on credibility assessment to establish model risk and credibility before its application (such as this study), since the conclusions of the study seem to be clinical in nature.

3. Material model – Does the model account for hysteresis and viscoelasticity? It would be interesting to see if the model can simulate progression of tears with repeated cyclic loading as observed in practise.

4. PL1 and PL2 in Figure 3 – I can not find the definition of these models in the methods section. What is the difference between these two? Geometry of the tears?

5. Material model of degeneration – ITM and k2 parameters. As I mentioned above, even if they are published elsewhere, context of these parameters is essential to understand how they influence the model. Is there any past evidence that the degenerated discs exhibit higher stiffness at larger strains?

6. Table 1 – Nucleus [29] [12]. Can the authors clarify what parameters were taken from [29]? This study does not talk about degeneration models. Is there a reason for the choice of 2MPa and 3MPa for mild and moderate cases?

7. 4Nm load – Is there a reason why the authors did not opt for the standard 7.5Nm load?

Perhaps a brief explanation in many of the above points would largely alleviate my concerns.

Results:

1. “information about the location of stress concentration can assist in identifying the site of discogenic pain [31].” overstates the evidence. To my knowledge, pain is a biopsychosocial phenomenon and cannot be inferred solely from stress maps produced by finite-element models. I recommend the authors modify this statement and cite more recent critical literature.

2. Use of Tresca criterion – this justification could be moved to the methods section.

3. Figure 10, 13, 16 – X axis label and Legend is missing. Data is incomplete.

4. It would also be interesting to see the trends in axial rotation (AR).

5. Polar plots – These plots are particularly interesting to see. However, it is hard to differentiate between mild and moderate. Perhaps use a different color.

6. Figure 18, 19, 20 – there is a mismatch between the sub plot titles a, b and the figure title. Flexion spelling in the sub plot.

7. “Stress relaxing effect” – I suggest the authors use another word to describe the notion of insignificant increases in stress magnitude as degeneration progresses throughout the manuscript. Because “stress relaxation” is a viscoelastic phenomenon and not to be misinterpreted in the context of this study.

8. Effect of location of tears – “The first major observation here is the striking rise in stress by 2 to 3 times in the case of posterior tears compared to other tears under all conditions of annulus degenerations.” – I can not follow this statement as the plots show the rise only in flexion loading.

9. ROM changes – It would be interesting to see how the moment-rotation plots vary with degeneration and tear location. Is there any effect on neutral and extension zones due to these model changes? Perhaps the authors can include these plots in the appendix if not in the main body.

10. Did the authors consider conducting any statistical tests to quantify the statistical significance of degeneration and tear location? This is highly recommended.

Discussion:

1. Clinical implications – ‘Flexion has increased risk of posterior tears; extension – anterior tears; lateral bending – lateral tears’ These qualitative inferences can be directly made solely by studying the geometry of tears from scans. But, after this numerical study, can any quantitative inferences be made? Such as, flexion above x degrees may be deteriorating in case of posterior tears etc.

2. “The mechanical behaviours reported here offer practical guidance on physical activity tailored for different age groups and tear conditions to mitigate damage and promote disc healing, thereby linking biomechanical insights with potential clinical strategies.” – This is an overstatement considering the above raised points and mentioned limitations of the study. Arguably, a much more comprehensive VVUQ study is needed to use this model as evidence in any clinical setting. I suggest the authors to rework these claims.

3. 75% tear length – Are the model results sensitive to this parameter? A graph indicating progression of stress/ROM with % length of tear may be included. Without this discussion, the choice of 75% seems arbitrary without any clinical reasoning.

References:

1. Please check all the references to align with journal formatting requirements (e.g. 27, 30)

Reviewer #2: This manuscript presents a finite element study aimed at calculating peak stresses resulting from radial annulus fibrosus tears at different locations, under different modes of loading, and different stages of degeneration. While the effect of increased stress at the ends of the tear, particularly in states where the tissue is stiffest, is intuitive, the manuscript does contribute to the literature with its thoroughness. The text of the manuscript is clear, but the conclusions rely heavily on interpretation of figures, which are not always used effectively.

- Table 1 is missing and Figures 10, 13, and 16 are empty.

- Please indicate Right and Left when applicable.

- Figure 8 shows an anterolateral stress peak that's on the order of the peak stresses around the tear. The reason for this should be clarified.

- The text refers in multiple places to the intact model, but these results are not shown. While it is intuitive that a tear will increase stress at a particular location, the manuscript is unclear on the magnitude of that increase.

- How do the peak Tresca stresses relate to ultimate strength of the AF tissue in each of the degeneration states?

**********

6. PLOS authors have the option to publish the peer review history of their article (what does this mean?). If published, this will include your full peer review and any attached files.

If you choose “no”, your identity will remain anonymous but your review may still be made public.

**Do you want your identity to be public for this peer review?**  For information about this choice, including consent withdrawal, please see our Privacy Policy.

Reviewer #1: **Yes:** Siril Teja Dukkipati, Ph.D.

Reviewer #2: No

**********

[NOTE: If reviewer comments were submitted as an attachment file, they will be attached to this email and accessible via the submission site. Please log into your account, locate the manuscript record, and check for the action link "View Attachments". If this link does not appear, there are no attachment files.]

To ensure your figures meet our technical requirements, please review our figure guidelines: https://journals.plos.org/plosone/s/figures

You may also use PLOS’s free figure tool, NAAS, to help you prepare publication quality figures: https://journals.plos.org/plosone/s/figures#loc-tools-for-figure-preparation.

NAAS will assess whether your figures meet our technical requirements by comparing each figure against our figure specifications.

# Author comment: Author response to Decision Letter 1

**DOI:** 10.1371/journal.pone.0352680.r002

31 Dec 2025

The responses to reviewers comments are uploaded in the document titled, "Response to Reviewers_R1.docx"

Attachment Submitted filename: Response to Reviewers_R1.docx

# Peer review: Decision Letter 1

**DOI:** 10.1371/journal.pone.0352680.r003

**Authors:** Alessandra Aldieri, Alessandra Aldieri

23 Feb 2026

PONE-D-25-46137R1

Progression of radial tears in L5-S1 intervertebral disc depends on location and type of movements: An in-silico study

PLOS One

Dear Dr. Bhat,

Thank you for submitting your manuscript to PLOS ONE. After careful consideration, we feel that it has merit but does not fully meet PLOS ONE’s publication criteria as it currently stands. Therefore, we invite you to submit a revised version of the manuscript that addresses the points raised during the review process.

Please make cahnges in Results and Discussion particularly according to the comments of one of the Reviewers, who wonders the clinical applicability of such a comparative and simplified study.

Please submit your revised manuscript by Apr 09 2026 11:59PM. If you will need more time than this to complete your revisions, please reply to this message or contact the journal office at plosone@plos.org. When you're ready to submit your revision, log on to https://www.editorialmanager.com/pone/ and select the 'Submissions Needing Revision' folder to locate your manuscript file.

Please include the following items when submitting your revised manuscript:

- A letter that responds to each point raised by the academic editor and reviewer(s). You should upload this letter as a separate file labeled 'Response to Reviewers'.
- A marked-up copy of your manuscript that highlights changes made to the original version. You should upload this as a separate file labeled 'Revised Manuscript with Track Changes'.
- An unmarked version of your revised paper without tracked changes. You should upload this as a separate file labeled 'Manuscript'.

-->If you would like to make changes to your financial disclosure, please include your updated statement in your cover letter. Guidelines for resubmitting your figure files are available below the reviewer comments at the end of this letter.

If applicable, we recommend that you deposit your laboratory protocols in protocols.io to enhance the reproducibility of your results. Protocols.io assigns your protocol its own identifier (DOI) so that it can be cited independently in the future. For instructions see: https://journals.plos.org/plosone/s/submission-guidelines#loc-laboratory-protocols. Additionally, PLOS ONE offers an option for publishing peer-reviewed Lab Protocol articles, which describe protocols hosted on protocols.io. Read more information on sharing protocols at https://plos.org/protocols?utm_medium=editorial-email&utm_source=authorletters&utm_campaign=protocols.

We look forward to receiving your revised manuscript.

Kind regards,

Alessandra Aldieri

Academic Editor

PLOS One

Journal Requirements:

1. If the reviewer comments include a recommendation to cite specific previously published works, please review and evaluate these publications to determine whether they are relevant and should be cited. There is no requirement to cite these works unless the editor has indicated otherwise.

2. Please review your reference list to ensure that it is complete and correct. If you have cited papers that have been retracted, please include the rationale for doing so in the manuscript text, or remove these references and replace them with relevant current references. Any changes to the reference list should be mentioned in the rebuttal letter that accompanies your revised manuscript. If you need to cite a retracted article, indicate the article’s retracted status in the References list and also include a citation and full reference for the retraction notice.

[Note: HTML markup is below. Please do not edit.]

Reviewers' comments:

Reviewer's Responses to Questions

**Comments to the Author**

1. If the authors have adequately addressed your comments raised in a previous round of review and you feel that this manuscript is now acceptable for publication, you may indicate that here to bypass the “Comments to the Author” section, enter your conflict of interest statement in the “Confidential to Editor” section, and submit your "Accept" recommendation.

Reviewer #2: All comments have been addressed

Reviewer #3: (No Response)

**********

2. Is the manuscript technically sound, and do the data support the conclusions?

The manuscript must describe a technically sound piece of scientific research with data that supports the conclusions. Experiments must have been conducted rigorously, with appropriate controls, replication, and sample sizes. The conclusions must be drawn appropriately based on the data presented.

Reviewer #2: Yes

Reviewer #3: Partly

**********

3. Has the statistical analysis been performed appropriately and rigorously?

Reviewer #2: Yes

Reviewer #3: N/A

**********

4. Have the authors made all data underlying the findings in their manuscript fully available?

The PLOS Data policy requires authors to make all data underlying the findings described in their manuscript fully available without restriction, with rare exception (please refer to the Data Availability Statement in the manuscript PDF file). The data should be provided as part of the manuscript or its supporting information, or deposited to a public repository. For example, in addition to summary statistics, the data points behind means, medians and variance measures should be available. If there are restrictions on publicly sharing data—e.g. participant privacy or use of data from a third party—those must be specified.

Reviewer #2: Yes

Reviewer #3: Yes

**********

5. Is the manuscript presented in an intelligible fashion and written in standard English?

PLOS ONE does not copyedit accepted manuscripts, so the language in submitted articles must be clear, correct, and unambiguous. Any typographical or grammatical errors should be corrected at revision, so please note any specific errors here.

Reviewer #2: Yes

Reviewer #3: Yes

**********

6. Review Comments to the Author

Please use the space provided to explain your answers to the questions above. You may also include additional comments for the author, including concerns about dual publication, research ethics, or publication ethics. (Please upload your review as an attachment if it exceeds 20,000 characters)

Reviewer #2: All of my comments from the prior review have been satisfactorily addressed in the revised manuscript.

Reviewer #3: GENERAL COMMENT

This paper presents on a computational study aimed to assess the effect of the presence and location of radial tears in the annulus fibrosus, and tissue quality, on the damage occurring to the annulus fibrosus under extension, flexion and bending moments.

The authors used a previously developed finite element model of the L5-S1 compartment, which was modified to simulate the effect of ageing on the material properties of the intervertebral disc. Three different versions of the model were thus generated, corresponding to a healthy, a mildly degenerated, and a moderately degenerated tissue.

For each model, different scenarios were tested by modifying the boundary conditions. Four different loading conditions were tested: pure flexion, pure extension and pure left/right lateral bending moment. Four different tear locations were simulated: anterior, posterior and posterolateral (PL1 and PL2). The output of the simulations was compared in terms of maximum Tresca stress.

The manuscript has significantly improved upon the first round of revisions, as the authors carefully addressed most of the reviewers’ comments. However, I have some concerns on how the results are presented and interpreted.

In light of the modelling choices (applied loads and material properties of the tissues) and the nature of the work that the authors themselves label as comparative and exploratory, I struggle to see any direct clinical implications of this work. I suggest the authors to revise the discussion section, by tuning down some statements, and to refrain from providing clinical suggestions based on the presented results.

Furthermore, to strengthen the work, it would be good to see some numbers accompanying the text in the results section. Although direct comparisons to clinical and/or normative healthy data may not be possible, reading the values would help appreciating the differences between tested conditions. Last, the quality of the figure could be improved to facilitate qualitative comparisons.

Please see my specific comments below.

SPECIFIC COMMENTS

- Methods -

1. Please clarify the link between the applied boundary conditions and real loading conditions. Are human intervertebral discs commonly experiencing those types of loads (pure moments) and to the same extent (4 Nm), during activities of daily living? In other words, how representative of in vivo loads are the implemented boundary conditions?

2. “Boundary conditions were designed to simulate four physiological movements: flexion, extension, left lateral bending, and right lateral bending, in the presence of a compressive load. A bending moment of 4 Nm was applied to a central reference point on the superior endplate of the L5 vertebra in all simulations.”

COMMENT: Please clarify where and how the compressive load was applied.

- Results -

3. Please add numbers/values (e.g. for the peak stresses) directly in the text of the results section to provide some quantitative assessment.

Discussion

4. As mentioned in the general comment, considering the nature of the work (exploratory and comparative, as acknowledged by the authors) and specific modelling choices, it is hard to see direct clinical implications of this study. Please revise the subsection ‘Clinical relevance of the results’, starting from the opening sentence, to reflect this.

5. “However, extension may be unsafe for older individuals, as degeneration increases the stress in the posterior and posterolateral region, which can further damage surrounding tissues and accelerate tear growth.”

COMMENT: In the absence of normative data to compare the reported results with, one cannot conclude whether the observed increase can be dangerous or not.

6. “Therefore, lateral bending motions should be strictly avoided for patients with posterolateral tears, regardless of age, which corroborates clinical findings [50].”

COMMENT: Please refrain from providing clinical indications. The presented results do not support such statements. For instance, it is not clear whether the applied moments – that differ from what is commonly used in similar computational studies - reflect any specific habitual conditions (e.g., activities of daily living or physiotherapy exercises). In addition, ref 50 does not seem appropriate.

7. “The clinical translation of techniques such as annular sealants, still under development, offers promise for treating such injuries earlier and more effectively [51]–[53].”

COMMENT: This whole sentence appears out of context, and should be removed.

8. In the limitations section, please state each limitation first and then explain what could be done differently and why you did it the way you did.

- Conclusion -

9. It is important to emphasize here that this is an exploratory and comparative study.

10. The term ageing was used only once before in text, in the introduction. It may not be clear to the readership what the authors are referring to here. Be consistent with the terminology and refrain from using keywords only in the introduction and conclusions sections, for sake of clarity. It may be worth, throughout the manuscript, to remind the readers that the effect of ageing was implemented by increasing the stiffness of the annulus fibrosus (i.e., mildly degenerated, and moderately degenerated states).

11. “This indicated that the presence of these tears could essentially be healed by conservative treatments of physiotherapy.”

COMMENT: The presented data does not support any such statements.

- Figures -

12. Please ensure that the various subplots in the figures use the same scales (i.e., same y limits for the colormap), otherwise the pictorial representation and visual information can be misleading (this is valid for both contours and radar plots).

13. Figure 28 could be improved by grouping the results in different subplots (or at least by showing the two parts - a and b – above each other) and should be moved to the Results section. All the results (and figures) should be introduced before the discussion.

**********

7. PLOS authors have the option to publish the peer review history of their article (what does this mean?). If published, this will include your full peer review and any attached files.

If you choose “no”, your identity will remain anonymous but your review may still be made public.

**Do you want your identity to be public for this peer review?**  For information about this choice, including consent withdrawal, please see our Privacy Policy.

Reviewer #2: **Yes:** Art Michalek

Reviewer #3: No

**********

[NOTE: If reviewer comments were submitted as an attachment file, they will be attached to this email and accessible via the submission site. Please log into your account, locate the manuscript record, and check for the action link "View Attachments". If this link does not appear, there are no attachment files.]

To ensure your figures meet our technical requirements, please review our figure guidelines: https://journals.plos.org/plosone/s/figures

You may also use PLOS’s free figure tool, NAAS, to help you prepare publication quality figures: https://journals.plos.org/plosone/s/figures#loc-tools-for-figure-preparation.

NAAS will assess whether your figures meet our technical requirements by comparing each figure against our figure specifications.

# Author comment: Author response to Decision Letter 2

**DOI:** 10.1371/journal.pone.0352680.r004

25 Mar 2026

The responses to reviewers' comments are provided in the uploaded document titled, "Response to Reviewers_R2.docx"

Attachment Submitted filename: Response to Reviewers_R2.docx

# Peer review: Decision Letter 2

**DOI:** 10.1371/journal.pone.0352680.r005

**Authors:** Alessandra Aldieri, Alessandra Aldieri, Alessandra Aldieri

14 Jun 2026

Progression of radial tears in L5-S1 intervertebral disc depends on location and type of movements: An in-silico study

PONE-D-25-46137R2

Dear Dr. Bhat,

We’re pleased to inform you that your manuscript has been judged scientifically suitable for publication and will be formally accepted for publication once it meets all outstanding technical requirements.

Within one week, you’ll receive an e-mail detailing the required amendments. When these have been addressed, you’ll receive a formal acceptance letter and your manuscript will be scheduled for publication.

An invoice will be generated when your article is formally accepted. Please note, if your institution has a publishing partnership with PLOS and your article meets the relevant criteria, all or part of your publication costs will be covered. Please make sure your user information is up-to-date by logging into Editorial Manager at Editorial Manager® and clicking the ‘Update My Information' link at the top of the page. For questions related to billing, please contact billing support.

If your institution or institutions have a press office, please notify them about your upcoming paper to help maximize its impact. If they’ll be preparing press materials, please inform our press team as soon as possible -- no later than 48 hours after receiving the formal acceptance. Your manuscript will remain under strict press embargo until 2 pm Eastern Time on the date of publication. For more information, please contact onepress@plos.org.

Kind regards,

Alessandra Aldieri

Academic Editor

PLOS One

Additional Editor Comments (optional):

Reviewers' comments:

Reviewer's Responses to Questions

**Comments to the Author**

1. If the authors have adequately addressed your comments raised in a previous round of review and you feel that this manuscript is now acceptable for publication, you may indicate that here to bypass the “Comments to the Author” section, enter your conflict of interest statement in the “Confidential to Editor” section, and submit your "Accept" recommendation.

Reviewer #2: All comments have been addressed

Reviewer #3: All comments have been addressed

**********

2. Is the manuscript technically sound, and do the data support the conclusions?

The manuscript must describe a technically sound piece of scientific research with data that supports the conclusions. Experiments must have been conducted rigorously, with appropriate controls, replication, and sample sizes. The conclusions must be drawn appropriately based on the data presented.

Reviewer #2: (No Response)

Reviewer #3: Yes

**********

3. Has the statistical analysis been performed appropriately and rigorously?

Reviewer #2: (No Response)

Reviewer #3: N/A

**********

4. Have the authors made all data underlying the findings in their manuscript fully available?

The PLOS Data policy requires authors to make all data underlying the findings described in their manuscript fully available without restriction, with rare exception (please refer to the Data Availability Statement in the manuscript PDF file). The data should be provided as part of the manuscript or its supporting information, or deposited to a public repository. For example, in addition to summary statistics, the data points behind means, medians and variance measures should be available. If there are restrictions on publicly sharing data—e.g. participant privacy or use of data from a third party—those must be specified.

Reviewer #2: (No Response)

Reviewer #3: Yes

**********

5. Is the manuscript presented in an intelligible fashion and written in standard English?

PLOS ONE does not copyedit accepted manuscripts, so the language in submitted articles must be clear, correct, and unambiguous. Any typographical or grammatical errors should be corrected at revision, so please note any specific errors here.

Reviewer #2: (No Response)

Reviewer #3: Yes

**********

6. Review Comments to the Author

Please use the space provided to explain your answers to the questions above. You may also include additional comments for the author, including concerns about dual publication, research ethics, or publication ethics. (Please upload your review as an attachment if it exceeds 20,000 characters)

Reviewer #2: (No Response)

Reviewer #3: I would like to thank the authors for addressing all my previous comments.

I believe that the manuscript has improved in clarity after the last round of revisions.

I have few minor comments left, which are listed below:

1) What was the IEC approval required for?

COMMENT: This is just a curiosity of mine, considering that this was a purely computational study, which employed a previously developed model.

2) At page 6, the authors write: "To represent the experimentally observed [...] by 50% and 100%, respectively".

COMMENT: Please add some references to the cited work, for the readers to more easily access that information.

3) Page 32, Effect of degeneration on range of motion (ROM)

COMMENT: Please provide the approximate ROM for the different tested cases, for sake of clarity.

**********

7. PLOS authors have the option to publish the peer review history of their article (what does this mean?). If published, this will include your full peer review and any attached files.

If you choose “no”, your identity will remain anonymous but your review may still be made public.

**Do you want your identity to be public for this peer review?**  For information about this choice, including consent withdrawal, please see our Privacy Policy.

Reviewer #2: No

Reviewer #3: No

**********

# Editor report: Acceptance letter

**DOI:** 10.1371/journal.pone.0352680.r006

**Authors:** Alessandra Aldieri, Alessandra Aldieri, Alessandra Aldieri

PONE-D-25-46137R2

PLOS One

Dear Dr. Bhat,

I'm pleased to inform you that your manuscript has been deemed suitable for publication in PLOS One. Congratulations! Your manuscript is now being handed over to our production team.

At this stage, our production department will prepare your paper for publication. This includes ensuring the following:

* All references, tables, and figures are properly cited

* All relevant supporting information is included in the manuscript submission,

* There are no issues that prevent the paper from being properly typeset

You will receive further instructions from the production team, including instructions on how to review your proof when it is ready. Please keep in mind that we are working through a large volume of accepted articles, so please give us a few days to review your paper and let you know the next and final steps.

Lastly, if your institution or institutions have a press office, please let them know about your upcoming paper now to help maximize its impact. If they'll be preparing press materials, please inform our press team within the next 48 hours. Your manuscript will remain under strict press embargo until 2 pm Eastern Time on the date of publication. For more information, please contact onepress@plos.org.

You will receive an invoice from PLOS for your publication fee after your manuscript has reached the completed accept phase. If you receive an email requesting payment before acceptance or for any other service, this may be a phishing scheme. Learn how to identify phishing emails and protect your accounts at https://explore.plos.org/phishing.

If we can help with anything else, please email us at customercare@plos.org.

Thank you for submitting your work to PLOS ONE and supporting open access.

Kind regards,

PLOS ONE Editorial Office Staff

on behalf of

Dr. Alessandra Aldieri

Academic Editor

PLOS One
